# Synthesis, Thermal, Structural Analyses, and Photoluminescent Properties of a New Family of Malonate-Containing Lanthanide(III) Coordination Polymers

**DOI:** 10.3389/fchem.2019.00260

**Published:** 2019-04-30

**Authors:** Sajjad Hussain, Xuenian Chen, William T. A. Harrison, Saeed Ahmad, Mark R. J. Elsegood, Islam Ullah Khan, Shabbir Muhammad

**Affiliations:** ^1^Henan Key Laboratory of Boron Chemistry and Advanced Energy Materials, School of Chemistry and Chemical Engineering, Henan Normal University, Xinxiang, China; ^2^Department of Chemistry, Mohi Ud Din Islamic University Nerian Sharif, Azad Jammu and Kashmir, Pakistan; ^3^College of Chemistry and Molecular Engineering, Zhengzhou University, Zhengzhou, China; ^4^Department of Chemistry, University of Aberdeen, Aberdeen, Scotland; ^5^Department of Chemistry, College of Sciences and Humanities, Prince Sattam Bin Abdulaziz University, Al-Kharj, Saudi Arabia; ^6^Chemistry Department, Loughborough University, Loughborough, United Kingdom; ^7^Department of Chemistry, Government College University, Lahore, Pakistan; ^8^Department of Physics, College of Science, King Khalid University, Abha, Saudi Arabia

**Keywords:** lanthanides, malonate, photoluminescence, crystal structure, optoelectronic properties

## Abstract

Five new Lanthanide(III) complexes of malonic acid (HOOC-CH_2_-COOH); {[Gd(C_3_H_2_O_4_)(H_2_O)_4_]·NO_3_}_*n*_ (**1**), {[Tb(C_3_H_2_O_4_)(H_2_O)_4_]·NO_3_}_*n*_ (**2**), {[Ho(C_3_H_2_O_4_)(H_2_O)_4_]·NO_3_}_*n*_ (**3**), [Er(C_3_H_2_O_4_)(C_3_H_3_O_4_)(H_2_O)_2_]_*n*_ (**4**), and {[Eu_2_(C_3_H_2_O_4_)_2_(C_3_H_3_O_4_)_2_(H_2_O)_6_]·4H_2_O}_*n*_ (**5**) were synthesized and characterized by elemental, infrared spectral, and thermal analyses. The structures of compounds **1**–**5** were determined by single crystal X-ray diffraction technique. The X-ray analysis reveals that compounds **1**, **2**, and **3** are isostructural and crystallized in the orthorhombic space group *Pmn*2_1_. The lanthanide(III) ions are coordinated by four carboxylate and four water oxygen atoms adopting a distorted square antiprism geometry. The LnO_8_ square antiprisms are linked into infinite layers by malonate (C_3_H_2_${\text{O}}_{4}^{2-}$) dianions sandwiching sheets of nitrate counter ions. Compound **4** contains ErO_8_ square antiprisms linked into a two-dimensional network by hydrogen malonate (C_3_H_3_${\text{O}}_{4}^{-}$) anions and malonate dianions. The europium complex, **5** is dinuclear having the two europium(III) ions (Eu1 and Eu2) bridged by carboxylate groups of hydrogen malonate ligands. The europium ions in **5** are nine-coordinate and exhibit a distorted monocapped square antiprism geometry. All the structures are consolidated by O–H⋯ O hydrogen bonds. The photoluminescence spectra of **1–5** exhibit characteristic emissions in the visible region. The IR spectra and thermal data are consistent with the structural results. The room-temperature effective magnetic moments for **1–4** are in good agreement with those expected for the free ions, while the data for **5** indicates that low-lying excited states contribute to the observed moment. The compound **1** was further subjected to quantum computational calculations to explore its optoelectronic properties including; density of states (DOS), dielectric function, refractive index, extinction coefficient, and absorption spectrum, to highlight the possible applications of such materials in the optoelectronics.

**Graphical Abstract:**
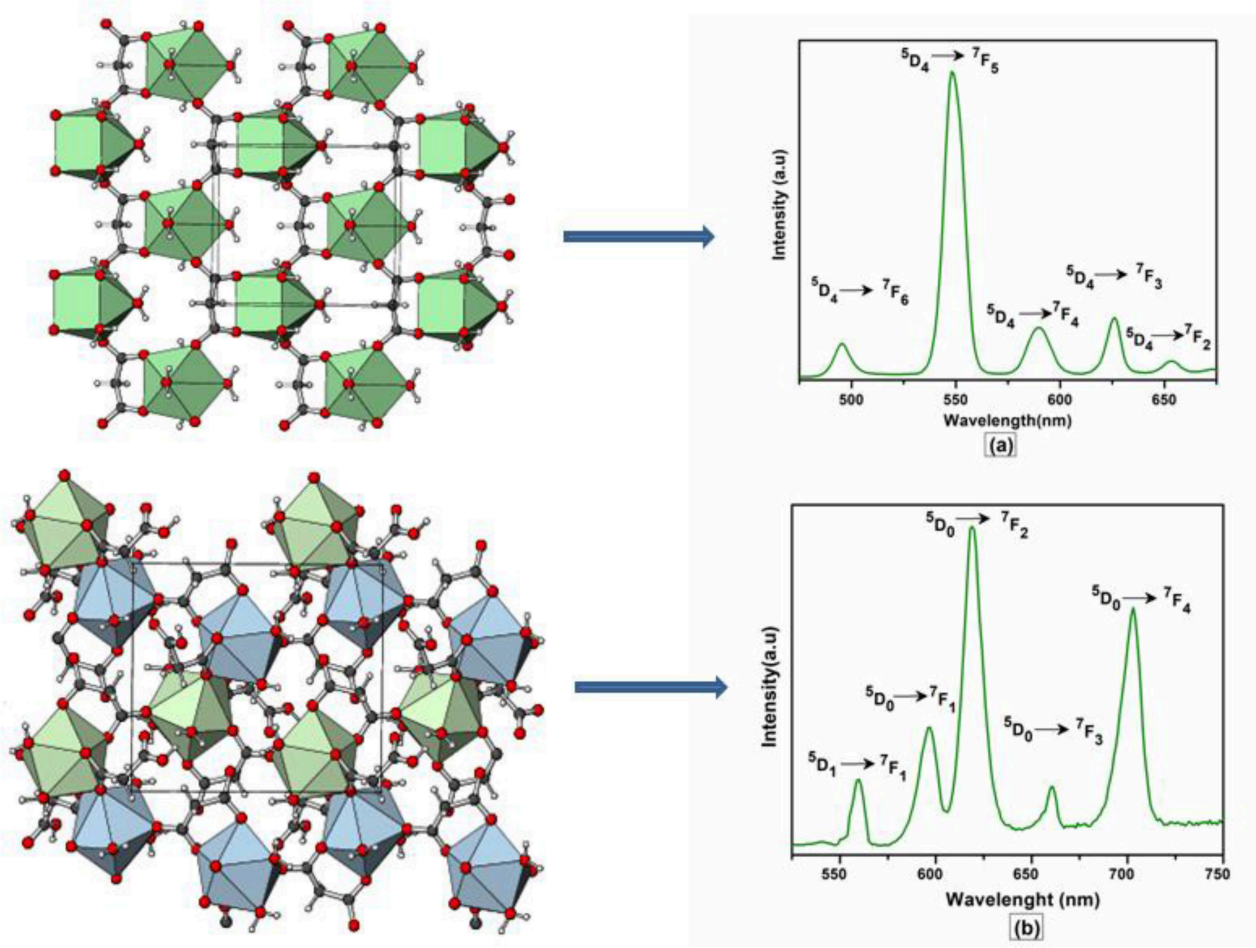


## Introduction

In the modern hi-tech society, lanthanide-based metal-organic frameworks find a wide range of applications in several cutting-edge scientific fields, such as contrast agents (Caravan et al., 1999; Wahsner et al., 2019), catalysis (Shibasaki and Yoshikawa, 2002; He et al., 2013; Pagis et al., 2016), gas storage and purification (Reineke et al., 1999; He et al., 2013; Roy et al., 2014), magnetism (Woodruff et al., 2013; Zhu et al., 2016; Gao et al., 2018), and optoelectronic devices (Kenyon, 2002; Armelao et al., 2010; Heffern et al., 2013). The study of photo physical properties of lanthanides (Ln) triggered the potential use of these compounds not only in color televisions and fluorescent tubes but also in optical amplifiers, luminescent solar concentrators, active waveguides, organic light emitting diodes, and immunoassays (Bünzli, 2010; Katkova and Bochkarev, 2010; Heffern et al., 2013; Reisfeld, 2015). The optoelectronic properties of lanthanide complexes originate from the inner *f-f* electron transitions. The shielding of 4*f* orbitals confers the excellent luminescence properties of Ln^3+^ ions (Reisfeld and Jorgensen, 2012; Heffern et al., 2013; Reisfeld, 2015). Several reports have appeared in the literature on the physical and optical properties of lanthanide complexes, which demonstrate their significant technological interest (Kenyon, 2002; Faulkner et al., 2005; Terai et al., 2006; Daiguebonne et al., 2008; Dos Santos et al., 2008; Armelao et al., 2010; Zhuravlev et al., 2011; Räsänen et al., 2014; Buenzli, 2015; Sharma and Narula, 2015; Sun et al., 2015; George et al., 2016).

Lanthanide(III) ions are hard Lewis acids and prefer to combine with hard Lewis bases such as oxygen donors (Bünzli, 2014). On the basis of this fact, carboxylate ligands have been widely applied for the preparation of lanthanide coordination polymers (Hansson, 1973a,b; Wenmei et al., 1992; Marrot and Trombe, 1994; Benmerad et al., 2000; Hernández-Molina et al., 2000, 2002, 2003; Doreswamy et al., 2003, 2005; Thirumurugan and Natarajan, 2004; Cui et al., 2005; Yan et al., 2005; Cañadillas-Delgado et al., 2006; Deacon et al., 2006; Rahahlia et al., 2007; Zhang et al., 2007; Fang et al., 2008; Wang et al., 2009; Chrysomallidou et al., 2010; Silva et al., 2010; Jin et al., 2012; Seidel et al., 2012; Sharif et al., 2012; Calahorro et al., 2013; Tian et al., 2013; Bünzli, 2014; Delgado et al., 2016; Li et al., 2017). The flexible aliphatic dicarboxylates (e.g., malonate and succinate) are more fascinating than the rigid mono or aromatic carboxylates due to many possible conformations (Cui et al., 2005; Rahahlia et al., 2007; Wang et al., 2009; Delgado et al., 2016). The use of multidentate organic linkers together with the high coordination number of lanthanide ions help to construct diverse structural motifs with unexpected properties. The structural arrangements in the lanthanide dicarboxylate polymers range from one- (Yan et al., 2005; Chrysomallidou et al., 2010; Silva et al., 2010; Li et al., 2017), two- (Hernández-Molina et al., 2000; Cañadillas-Delgado et al., 2006; Li et al., 2017), to three-dimensional (Hansson, 1973a,b; Wenmei et al., 1992; Marrot and Trombe, 1994; Benmerad et al., 2000; Hernández-Molina et al., 2002, 2003; Doreswamy et al., 2003, 2005; Thirumurugan and Natarajan, 2004; Cañadillas-Delgado et al., 2006; Zhang et al., 2007; Fang et al., 2008; Chrysomallidou et al., 2010; Jin et al., 2012; Seidel et al., 2012; Calahorro et al., 2013) coordination networks. Malonic acid, C_3_H_4_O_4_, is a simple dicarboxylic acid that exhibits a rather flexible stereochemistry and a variety of coordination modes toward metal ions. It can coordinate as a monodentate, bridging, and chelating ligand in monoanionic (hydrogen malonate, C_3_H_3_${\text{O}}_{4}^{-}$) or dianionic (malonate, C_3_H_2_${\text{O}}_{4}^{2-}$) form (Rodríguez-Martín et al., 2002; Delgado et al., 2016). A number of reports have been published on the synthesis and structures of malonate-containing lanthanide polymers, which describe the versatility of the bonding modes of the malonate group (Hansson, 1973a,b; Wenmei et al., 1992; Marrot and Trombe, 1994; Benmerad et al., 2000; Hernández-Molina et al., 2000, 2002, 2003; Doreswamy et al., 2005; Cañadillas-Delgado et al., 2006; Zhang et al., 2007; Fang et al., 2008; Chrysomallidou et al., 2010; Silva et al., 2010; Jin et al., 2012; Delgado et al., 2016). Delgado et al. reported a detailed overview on the crystal structures and topologies of these systems, as well as the molecular structures assembled by hydrogen-bonding from low-dimensional entities to higher-dimensional supramolecular architectures. The study describes that most of the Ln^3+^-malonate (mal^2−^) complexes exist as dinuclear species (Delgado et al., 2016), while the reports about the mononuclear complexes are rare (Wenmei et al., 1992; Marrot and Trombe, 1994; Chrysomallidou et al., 2010; Silva et al., 2010).

In order to enhance the fundamental knowledge of structural chemistry of lanthanide-carboxylate frameworks and in view of our continuous interest in this direction (Hussain et al., 2014, 2015a,b, 2018), we report here the syntheses, characterization, crystal structures, photoluminescence, and magnetic properties of five novel lanthanide coordination polymers involving malonate ligand. They include; {[M(C_3_H_2_O_4_)(H_2_O)_4_]·NO_3_}_*n*_ (M = Gd, Tb, Ho) (**1**-**3**), [Er(C_3_H_2_O_4_)(C_3_H_3_O_4_)(H_2_O)_2_]_*n*_ (**4**), and {[Eu_2_(C_3_H_2_O_4_)_2_(C_3_H_3_O_4_)_2_(H_2_O)_6_]·4H_2_O}_*n*_ (**5**). The optoelectronic properties including density of states, dielectric function, refractive index, extinction coefficient, and absorption spectrum, were also investigated for complex **1** with the help of DFT calculations. Owing to the bigger size of lanthanide complexes, they are very difficult to deal quantum chemically. Nonetheless, there are some previous computational studies including the structures and reactions (Eisenstein and Maron, 2002), bonding characteristics (Adamo and Maldivi, 1997), determination of ligand-field parameters (Ishikawa et al., 2003), effective core potential studies (Cundari et al., 1995), and details of computational methods applied in lanthanide and actinide chemistry etc (Dolg, 2015). To the best of our knowledge, this is the first report that describes the formation of lanthanide complexes including a nitrate ion and dinuclear Eu^3+^ or Er^3+^ complexes containing a hydrogen malonate anion, and their photoluminescent properties.

## Experimental

### Reagents and Measurements

The nitrate salts of Gd, Tb, and Ho{M(NO_3_)_3_·6H_2_O}, ErCl_3_·6H_2_O, and EuCl_3_·6H_2_O were purchased from Alfa Aesar, a Johnson Matthey Company, USA. Malonic acid was obtained from Merck Chemical Co. Germany.

### Synthesis of Complexes

#### Synthesis of {[M(C_3_H_2_O_4_)(H_2_O)_4_]·NO_3_}_n_(M = Gd, Tb, Ho) (1–3)

The compounds 1–3 were synthesized by reacting 0.225 g (0.5 mmol) of M(NO_3_)_3_·6H_2_O (M = Gd, Tb, Ho) dissolved in 5 mL de-ionized water and 0.156 g (1.5 mmol) of malonic acid in 20 mL ethanol. The solutions were stirred for 2 h at room temperature. The pH of the reaction mixture was adjusted between 5 and 6 with 0.1 M NaOH solution. The solutions were filtered and kept at ambient temperature (or in the refrigerator) for crystallization. The crystals appeared in the solution after 2–3 weeks. The complexes **1, 2**, and **3** were obtained as light yellow, colorless, and light pink crystals, respectively. They were separated by vacuum filtration and rinsed with ethanol. Yield: ~50%.

#### Synthesis of [Er(C_3_H_2_O_4_)(C_3_H_3_O_4_)(H_2_O)_2_]_n_ (4)

Malonic acid (0.104 g, 1 mmol) and ErCl_3_·6H_2_O (0.137 g, 0.5 mmol) were separately dissolved in 20 mL ethanol and 10 mL deionized water, respectively. The solutions were mixed and stirred for 2 h in a round-bottom flask at room temperature. During stirring, 0.1 M NaOH solution was used to maintain the pH of the mixture between 5 and 6. After 10 days, pink crystals of **4** appeared in solution, which were isolated by filtration and rinsed with methanol. Yield: 49%.

#### Synthesis of {[Eu_2_(C_3_H_2_O_4_)_2_(C_3_H_3_O_4_)_2_(H_2_O)_6_]·4H_2_O}_n_ (5)

To a solution of 0.183 g (0.5 mmol) of EuCl_3_·6H_2_O in 10 mL deionized water was added 0.104 g (1 mmol) of malonic acid dissolved in 25 mL ethanol. The solutions were mixed in round bottom flask and stirred for 3 h at room temperature at a pH of 5–6 maintained by using 0.1 M NaOH solution. After 2 weeks, colorless crystals of **5** were recovered by filtration and rinsed with methanol. Yield: 43%.

##### Analytical and spectroscopic data

C_3_H_10_NO_11_Gd (1): Calc. (%) C 9.16, H 2.55, N 3.56; Found (%): C 9.25, H 2.59, N 3.50.IR (cm^−1^): ν = 3583, 3369 (O-H), 2927 (C-H), 1582 (COO)_as_, 1384,1370 (COO)_s_, 276 (Gd-O); δ = 1463, 1149 (C-H),953 (COO), 819 (NO_3_); ρ = 836 (H_2_O), 735 (CH_2_).

C_3_H_10_NO_11_Tb (2): Calc. (%) C 9.13; H 2.55; N 3.55; Found (%): C 9.25, H 2.62, N 3.45. IR (cm^−1^): ν = 3585, 3369 (O-H), 2926 (C-H), 1583 (COO)_as_, 1384,1370 (COO)_s_, 285 (Tb-O); δ = 1463, 1149 (C-H), 954 (COO), 819 (NO_3_); ρ = 837 (H_2_O), 735 (CH_2_).

C_3_H_10_NO_11_Ho (3): Calc. (%): C 8.96, H 2.49, N 3.49; Found (%): C 8.81, H 2.52, N 3.45. IR (cm^−1^): ν = 3591, 3369 (O-H), 2928 (C-H), 1586 (COO)_as_, 1384,1372 (COO)_s_, 290 (Ho-O); δ = 1463, 1149 (C-H), 955 (COO), 819 (NO_3_); ρ = 838 (H_2_O), 739 (CH_2_).

C_6_H_9_O_10_Er (4): Calc. (%): C 17.65, H 2.22; Found (%): C 17.01, H 2.11. IR (cm^−1^): ν = 3573, 3338 (O-H), 2921 (C-H), 1697, 1566 (COO)_as_, 1383, 1279 (COO)_s_; δ = 1449, 1186 (C-H), 967 (COO); ρ = 803 (H_2_O), 712 (CH_2_).

C_6_H_15_O_13_Eu (5): Calc. (%): C 16.11, H 3.38; Found (%): C 16.72, H 3.92. IR (cm^−1^): ν = 3401 (O-H), 1714, 1570 (COO)_as_, 1384(COO)_s_; δ = 1440, 1206 (C-H), 964 (COO); ρ = 711 (CH_2_). (Malonic acid, ν = 3398 (O-H), 2992, 2948 (C-H), 1737, 1706 (COO)_as_, 1418, 1398 (COO)_s_; δ = 1438, 1174 (C-H), 920 (COO); ρ = 804 (H_2_O), 771 (CH_2_)).

### Physical Measurements

Elemental analyses were carried out on a Varion Micro Cube, Elementar, Germany. FTIR spectra were recorded over the frequency range 4,000–250 cm^−1^ on a Perkin Elmer FTIR 180 spectrophotometer using KBr pellets. Thermal analyses were performed from room temperature to 1,000°C at heating rate of 10°C min^−1^ in air on a Thermo-gravimeter Analyzer/Differential Scanning Calorimeter model SDT Q 600 (TA Instruments, USA). The excitation and emission spectra were recorded on photoluminescence spectrometer FLS 180 (Edinburg Instruments). The magnetic susceptibility measurements were conducted on Evans balance (Sherwood Scientific Ltd. UK) at room temperature and Hg[Co(SCN)_4_] was used as a calibrant. The diamagnetic corrections for the component atoms were determined using Pascal constant (Kahn, 1993; Earnshaw, 2013) and details of the method is given the Supporting Information.

### Crystal Structure Determinations

Intensity data for compounds **1**–**5** were collected on a Bruker APEXII CCD diffractometer at 150 K using MoKα radiation (λ = 0.71073 Å). An empirical absorption correction was carried out using SADABS (Sheldrick, 2014). The crystal structures were solved by direct methods with SHELXS-97 (Sheldrick, 2008) and refined by full-matrix least-squares on *F*^2^ using SHELXL-2014 (Sheldrick, 2015). For molecular graphics, ORTEP 3 was used (Farrugia, 2012). The C-bound H atoms were geometrically placed (C–H = 0.99 Å) and refined as riding atoms. The O-bound H atoms were located in difference maps and refined freely as riding atoms in their as-found relative positions or with gentle restrains. One of the water molecules of crystallization in **5** is disordered over two adjacent locations and its H atoms could not be located. The constraint *U*iso(H) = 1.2*U*_eq_(carrier) was applied in most of the cases. Details of the data collection and refinement details are summarized in Table 1.

**Table 1:** Crystal data for compounds **1–5**.

	**1**	**2**	**3**	**4**	**5**
Empirical formula	C_3_H_10_NO_11_Gd	C_3_H_10_NO_11_Tb	C_3_H_10_NO_11_Ho	C_6_H_9_O_10_Er	C_6_H_15_O_13_Eu
*M*_r_	393.37	395.04	401.05	408.39	447.14
*T* (K)	150(2)	150(2)	150(2)	150(2)	150(2)
λ (Å)	0.71073	0.71073	0.71073	0.71073	0.71073
Crystal system	Orthorhombic	Orthorhombic	Orthorhombic	Orthorhombic	Monoclinic
Space group	*Pmn*2_1_ (No. 31)	*Pmn*2_1_ (No. 31)	*Pmn*2_1_ (No. 31)	*Fdd*2 (No. 43)	*P*2_1_/*n* (No. 14)
*a* (Å)	8.0955 (8)	8.0863 (6)	8.0652 (12)	15.2618 (16)	10.7945 (5)
*b* (Å)	6.7470 (6)	6.7359 (5)	6.7123 (9)	10.0290 (10)	12.3934 (6)
*c* (Å)	9.3030 (9)	9.2562 (7)	9.1895 (11)	13.9992 (15)	20.2839 (10)
β (°)	90	90	90	90	100.3900 (7)
*V* (Å^3^)	508.13 (8)	504.17 (7)	497.48 (12)	2142.7 (4)	2669.1 (2)
*Z*	2	2	2	8	8
*F*(000)	374	376	380	1,544	1,744
ρ_calc_ (g cm^−3^)	2.571	2.602	2.677	2.532	2.225
μ (mm^−1^)	6.58	7.07	8.01	7.88	4.77
Total data, 2θ_max_ (°)	5901, 63.8	5802, 61.2	5384, 61.2	6299, 63.8	31194, 61.2
*R*_Int_	0.031	0.020	0.025	0.035	0.036
Merged, observed [*I*> 2σ(*I*)] data	1747, 1732	1642, 1635	1612, 1594	1742, 1562	8180, 7533
*R*(*F*)[*I*> 2σ(*I*)]	0.018	0.012	0.016	0.019	0.025
*wR*(*F*^2^) (all data)	0.043	0.029	0.033	0.038	0.063
Absolute structure parameter	0.036 (11)	0.025 (15)	0.026 (9)	0.045 (12)	—
min., max.Δρ (*e* Å^−3^)	−1.79, +0.71	−0.83, +0.75	−0.49, +1.19	−1.12, +0.87	−1.55, +1.38
CCDC deposition number	1555502	1555503	1555504	1555505	1555506

## Results and Discussion

### Synthesis

The reaction of M(NO_3_)_3_·6H_2_O (M = Gd, Tb, Ho) with three equivalents of malonic acid (C_3_H_4_O_4_) in the presence of NaOH in water-ethanol medium afforded the crystals of {[M(C_3_H_2_O_4_)(H_2_O)_4_]·NO_3_}_n_ complexes (**1**–**3**). The presence of nitrate was not detected in any of the previously reported structures of lanthanide-malonate complexes. Similar reactions of MCl_3_·6H_2_O (M = Er, Eu) and malonic acid in 1:2 molar ratio gave the crystalline complexes [Er(C_3_H_2_O_4_)(C_3_H_3_O_4_)(H_2_O)_2_]_*n*_ (**4**) and {[Eu_2_(C_3_H_2_O_4_)_2_(C_3_H_3_O_4_)_2_(H_2_O)_6_]^.^4H_2_O}_*n*_ (**5**). The chloride ion did not participate in coordination demonstrating the greater affinity of Ln(III) ions for O than Cl. In every case, the pH of the reaction solution was adjusted to 5–6 in order to avoid the isolation of insoluble hydroxides. The compounds were isolated by slow evaporation of the reaction solution. The composition of the complexes was established from elemental and thermal analyses and verified by X-ray crystallography. All five complexes crystallized as polymeric substances.

### IR Spectroscopy

The FTIR spectra of **1–5** are shown in Figures S1–S5. In the IR spectrum of malonic acid two intense bands at 1,737 and 1,706 cm^−1^ are observed due to ν_as_(COO), while the symmetric stretches of carboxylate groups appear at 1,418 and 1,398 cm^−1^. The signals at 2,992, 2,948, and 3,398 cm^−1^ are associated with C-H and O-H stretching vibrations, respectively. The CH_2_ rock is detected at 771 cm^−1^.

In the IR spectra of all complexes the asymmetric and symmetric stretching bands of the carboxylate groups were observed around 1,600 and 1,300 cm^−1^, respectively (Deacon and Phillips, 1980; Rodríguez-Martín et al., 2002; Chrysomallidou et al., 2010; Hussain et al., 2018). There is a significant drop in frequencies of these bands relative to that of free malonic acid indicating the coordination of malonate ions. The broad peaks near 3,600 and 3,400 cm^−1^ correspond to the O-H stretching modes. The rocking vibration of coordinated H_2_O (ρ(H_2_O) was found at 837 cm^−1^ in complexes **1**–**3** and at 803 cm^−1^ in **4** and **5**. The medium band around 950 cm^−1^ is ascribed to bending vibration, δ(O–C–O) of the carboxylate group. The M-O bonds absorbed at 276, 285, and 290 cm^−1^ for complexes **1**, **2**, and **3**, respectively, which prabably represent the ν(M-O_water_) vibrations (Chrysomallidou et al., 2010). These absorptions confirmed the formation of complexes as they were not observed in the ligand spectra. The spectral results are in agreement with the reported literature (Marrot and Trombe, 1994; Doreswamy et al., 2003; Chrysomallidou et al., 2010; Mathew et al., 2012).

The bands at 819 cm^−1^ in **1**–**3** mark the presence of nitrate ions (Alhoshani et al., 2019). The absence of a band around 1,700 cm^−1^ for complexes **1**–**3** indicates the deprotonation of COOH and coordination of carboxylate dianions to the metal ions. However, the peaks at 1,697 and 1,714 cm^−1^ in **4** and **5**, respectively, correspond to C = O stretches of protonated carboxylic acid (COOH) (Chrysomallidou et al., 2010) as it is evident from crystal structures that the hydrogen malonate ions are present in these compounds.

### Thermal Analysis

**Figure 1:**
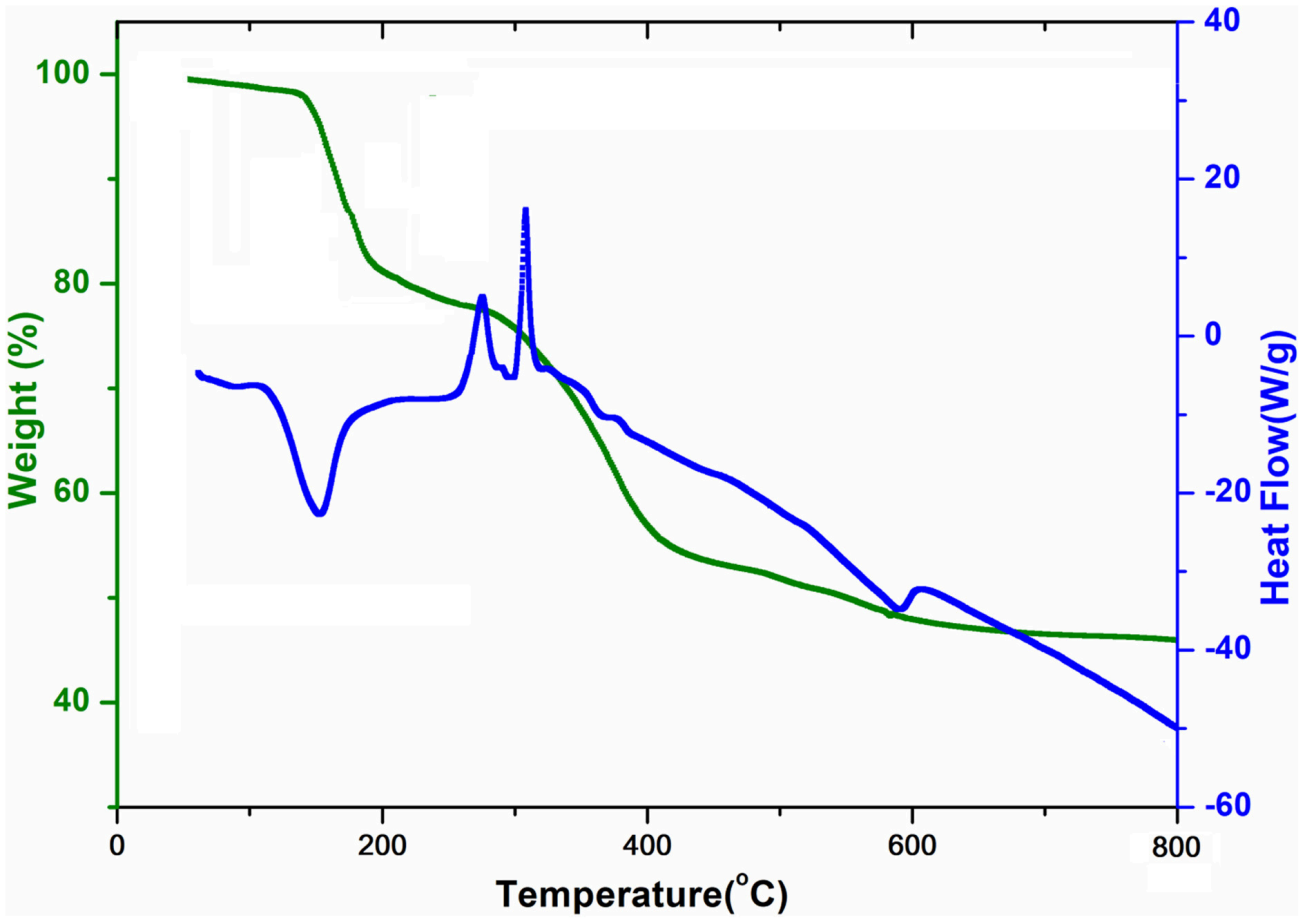
TGA curve and DSC curve of {[Gd(C_3_H_2_O_4_)(H_2_O)_4_]·NO_3_}_*n*_ (**1**).

The simultaneous TG-DSC curves for complex **1** are shown in Figure 1. These curves express the mass losses in three consecutive steps. The first decomposition step occurs between 100 and 200°C that is attributed to the departure of four coordinated water molecules (experimental weight loss = 19%, calculated = 18.3%). This is evidenced by an endothermic peak of DSC at about 160°C. After the removal of water, the complex does not show sufficient thermal stability and starts releasing malonate ligand around 200°C. The decomposition of the malonic group is completed at 400°C with a weight loss of about 27% (calculated value 25.9%). The combustion of malonate is accompanied by two exothermic transitions in DSC. In the third stage nitrate is lost (as a nitrogen oxide) in the range of 400–650°C leaving behind ½Gd_2_O_3_ as a residue (experimental = 46%, calculated = 46.1%). The DSC plot shows an endothermic dip at about 600 °C attributable to the removal of nitrate.

A close similarity to **1** is noted concerning the TG-DSC profiles of compounds **2** and **3**, presented in Figures S6, S7, respectively. However, instead of 650°C for **1**, the decomposition is completed at 900 and 600°C for **2** and **3**, respectively. The DSC curves show endothermic and exothermic peaks that all are in agreement with the mass losses observed in the TG curves. The final residues in case of **2** and **3** correspond to 46 % ½Tb_2_O_3_ (calculated = 46.3%) and 47% ½Ho_2_O_3_ (calculated value 47.1%), respectively. The similarity of the thermal patterns suggests that the decomposition mechanism is the same for the three compounds.

As illustrated in Figure S8, for compound **4** the first mass loss of 8% occurs between 140 and 170°C, attributed to dehydration (calculated weight loss = 8.8 % for the removal of two water molecules) and is associated with an endothermic peak at 168°C. The loss of water at relatively higher temperature indicates the absence of water molecules of crystallization in the complex. After exclusion of coordinated water molecules, the anhydrous compound is stable up to 370°C. The second weight loss of 45% occurs due to the removal of two malonic groups in the temperature range of 370–900°C (calculated 50.2%), leaving behind 47% ½Er_2_O_3_ as residue (calculated = 46.8%). The DSC curve depicts two exotherms presumably due to combustion of organic moieties at 400 and 625°C.

The thermal behavior of complex **5** is described in Figure S9. The decomposition begins with the loss of two water molecules of crystallization in the temperature range 90–130°C (experimental weight loss = 8%; calculated = 8.1%). The second weight loss of 12% was observed in the range of 150–200°C and is ascribed to the elimination of three water molecules coordinated with metal atom. Thus, the thermogram clearly distinguishes the presence of uncoordinated and coordinated water molecules in the complex. After 225°C, the malonate ligands are lost reducing the mass by 41% (calculated = 45.8%). The final residue is 39% that is associated with ½Eu_2_O_3_ (calculated 39.4%). In the DSC plot an endotherm at about 150°C corresponds to the dehydration step, while the exotherms at about 300 and 410°C represent the combustion of the malonate ligands. The shapes of the TGA curves for **1**–**5** are similar to those of the other malonate-containing Ln^3+^ complexes described in the literature (Muraishi et al., 1991; Doreswamy et al., 2003; Chrysomallidou et al., 2010; Delgado et al., 2016) suggesting that the stability of the complexes is comparable to the reported ones.

### Description of Crystal Structures

Single-crystal X-ray structural analysis revealed that compounds **1, 2**, and **3** crystallize in the polar, orthorhombic space group *Pmn*2_1_ and are isostructural. Therefore, complex **1** is taken as an example to present and discuss the structures in detail with any significant differences for **2** and **3** noted, where ever applicable. Selected bond distances and bond angles for **1** are presented in Table 2, while for **2**–**5** in Tables S1–S4, respectively. The hydrogen bonding details for **1**–**5** are given in Tables S5–S9, respectively.

**Table 2:** Selected bond lengths (Å), bond angles (°), and torsion angles (°) for **1**.

Gd1–O1	2.330(3)	O1–Gd1–O1^i^	115.8(2)
Gd1–O2^i^	2.378(3)	O1–Gd1–O2	77.37(11)
Gd1–O3	2.454(5)	O1–Gd1–O3	72.00(11)
Gd1–O4	2.415(3)	O2–Gd1–O3	80.41(13)
Gd1–O5	2.421(5)	O2–Gd1–O4	112.51(12)
C1–O1	1.245(4)	O4–Gd1–O3	144.61(9)
C1–O1	1.264(5)	O5–Gd1–O3	117.72(16)
O1–C1–C2–C1^ii^	125.1(4)	O2–C1–C2–C1 ii	−55.4(6)

*Symmetry codes: (i) x–½, 1–y, z–½; (ii) –x+2, y, z; (iii) x, y-1, z; (iv) –x+3/2, -y+2, z+1/2*.

**Figure 2:**
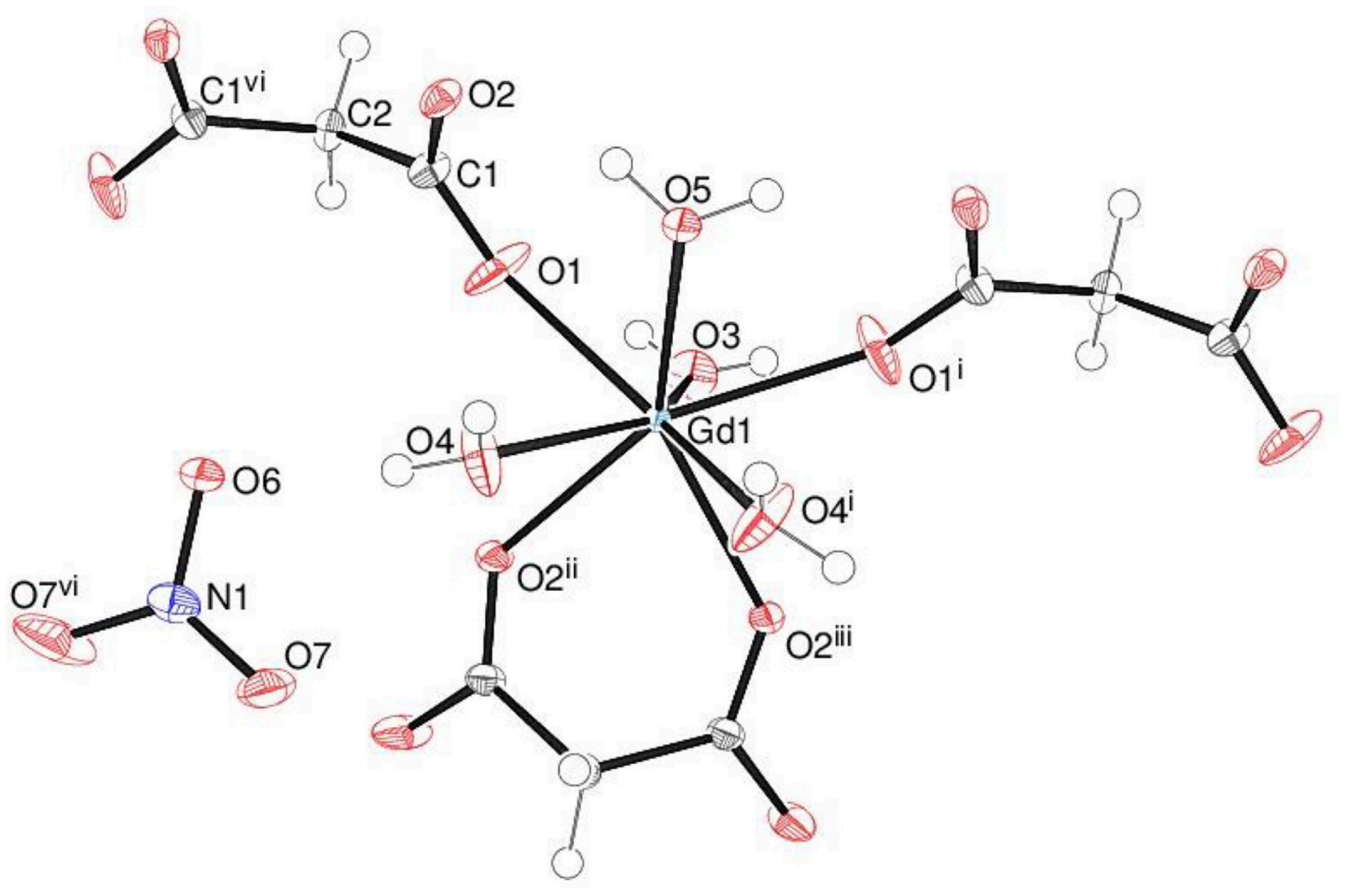
The asymmetric unit of **1** (50% displacement ellipsoids) expanded to show the full metal coordination sphere.

**Figure 3:**
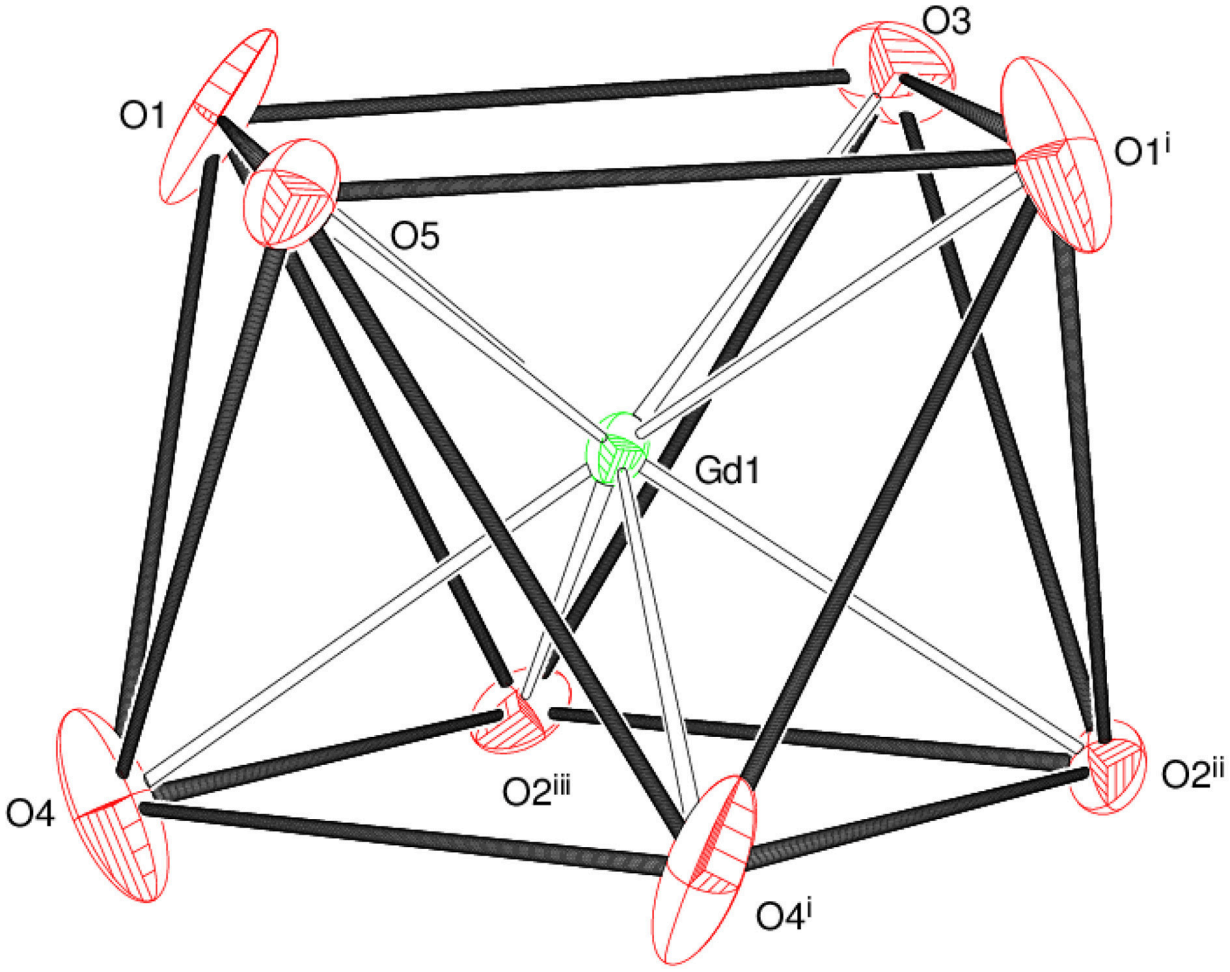
The square anti-prismatic metal coordination geometry in **1**.

The asymmetric unit of **1** shown in Figure 2 consists of a Gd^3+^ ion (site symmetry *m*), a malonate (C_3_H_2_${\text{O}}_{4}^{2-}$) ligand (with the central C atom lying on a crystallographic mirror plane), four water molecules (two lying on a mirror plane) and a nitrate counter ion (the N atom and one of the O atoms have *m* site symmetry). The cationic complex is polymeric with each Gd^3+^ ion coordinated by four O atoms from three different malonate groups and by four water molecules adopting a fairly regular square anti-prismatic geometry. Each square face of GdO_8_ coordination polyhedron consists of two malonate O atoms and two O atoms of water molecules, but their dispositions are different (Figure 3). In the O1/O1^i^/O3/O5 face, the water O atoms are “*trans”* (lying across the square diagonal), whereas in the other, O2^ii^/O2^iii^/O4/O4^i^, they are “*cis”* (adjacent). The angles subtended at the Gd atom by them vary in the range of 72.01 to 144.61°. The malonate ligand is bonded to three different symmetry-related metal atoms yielding a two dimensional coordination polymer. It forms a six-membered chelate ring with one Gd^3+^ ion, while the remaining two carboxyl oxygen atoms bind in a unidentate mode to the other two metal ions. In this way, the coordination mode of the bridging malonate group can be described as μ_3_-κ^2^O,O'κO”,κO”' (Delgado et al., 2016). The six-membered chelate ring is well described as a boat conformation, with C1 × 2 and O2 × 2 exactly coplanar by symmetry and C2 and Gd1 displaced in the same sense by 0.610 (6) Å and 0.623 (7) Å, respectively.

The mean Gd–O bond distance in **1** is 2.390(4) Å (Table 2). The comparable distances for Tb in **2** (Table S1) and for Ho in **3** (Table S2) are 2.377(3) and 2.352(4) Å, respectively. This trend agrees well with that expected in terms of the lanthanide contraction effect (Bünzli, 2014). In each case, the M–O_m_ (m = malonate) bonds are shorter than the M–O_w_ (w = water) bonds, which can be explained electrostatically in terms of stronger attraction to the delocalized negative charge on the malonate O atoms. The M-O bond lengths fall within the range observed for other Ln^3+^ complexes of malonate (Hernández-Molina et al., 2003; Cañadillas-Delgado et al., 2006; Delgado et al., 2016). The C1–O1 and C1–O2 bond lengths (Table 2) in the malonate ligand are almost equal, indicating delocalization of charge. The dihedral angle between the square planes is 2.84(16)° and the metal ion is displaced from the O1 and O2 planes by 1.248(2) and −1.330(2) Å, respectively.

**Figure 4:**
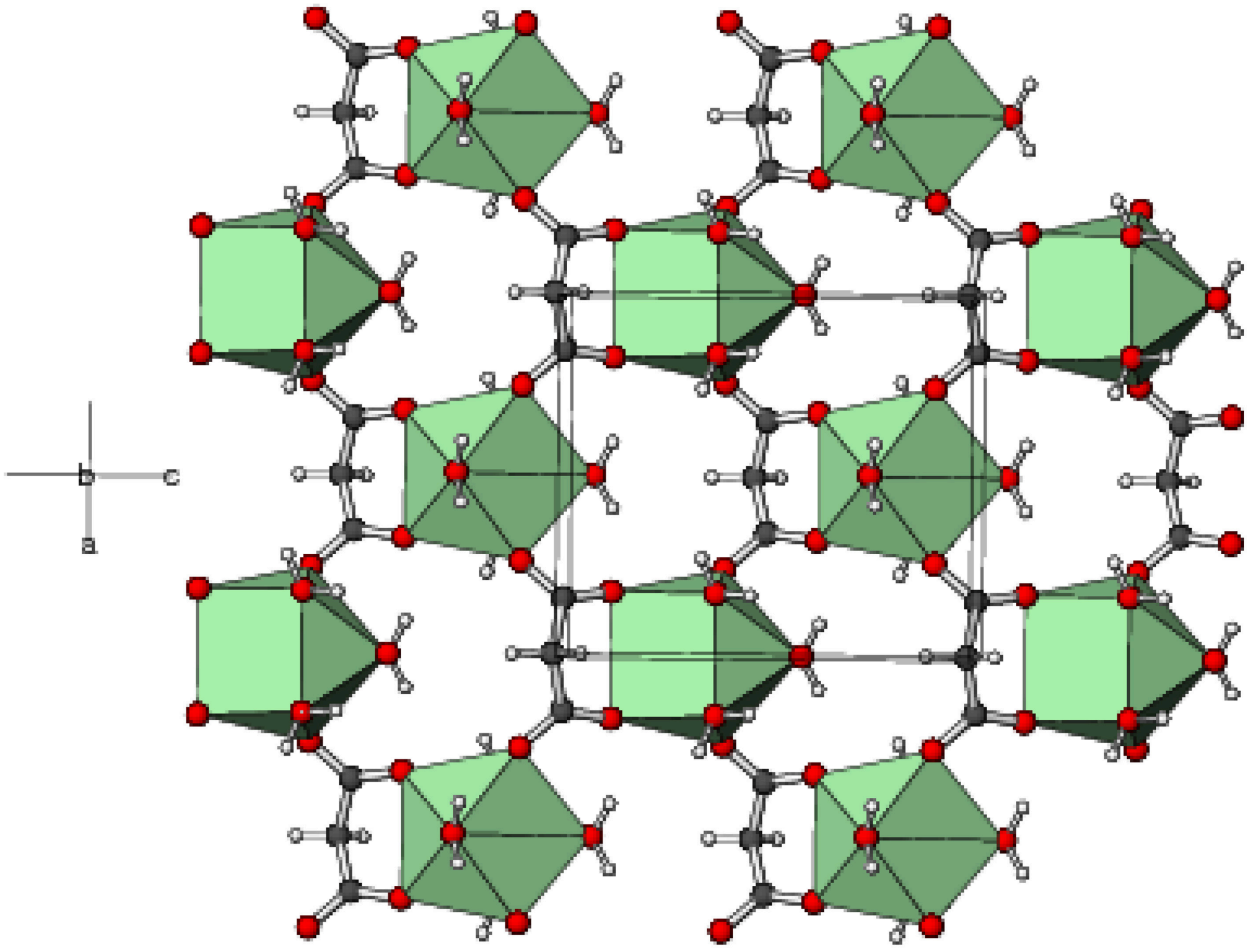
Part of a (010) layer in the structure of **1** with the GdO_8_ moieties represented as green polyhedra and C, H, and O atoms shown as dark grey, white, and red spheres, respectively.

In the extended structure of **1**, the ligands bridge the metal ions into infinite cationic sheets of the formula [Gd(C_3_H_2_O_4_)(H_2_O)_4_]^+^ propagating in the (010) plane (Figure 4). Each malonate ligand links with three metal ions and the shortest Gd⋯ Gd separation within a layer is 6.1705(9) Å, slightly less than the shortest inter-layer separation of 6.7470(7) Å [The corresponding data for **2** = 6.1498(7) and 6.7359(5) Å, respectively, and for **3** = 6.1179(11) and 6.7123(10) Å, respectively]. The nitrate counter ions occupy the inter-layer sites and the inter-sheet bonding is consolidated by O–H⋯ O hydrogen bonds (Table S5), with malonate O atoms, water molecules and nitrate-O atoms all acting as acceptors. The hydrogen-bonding patterns in the terbium (Table S6) and holmium (Table S7) compounds are essentially identical to that in **1**.

**Figure 5:**
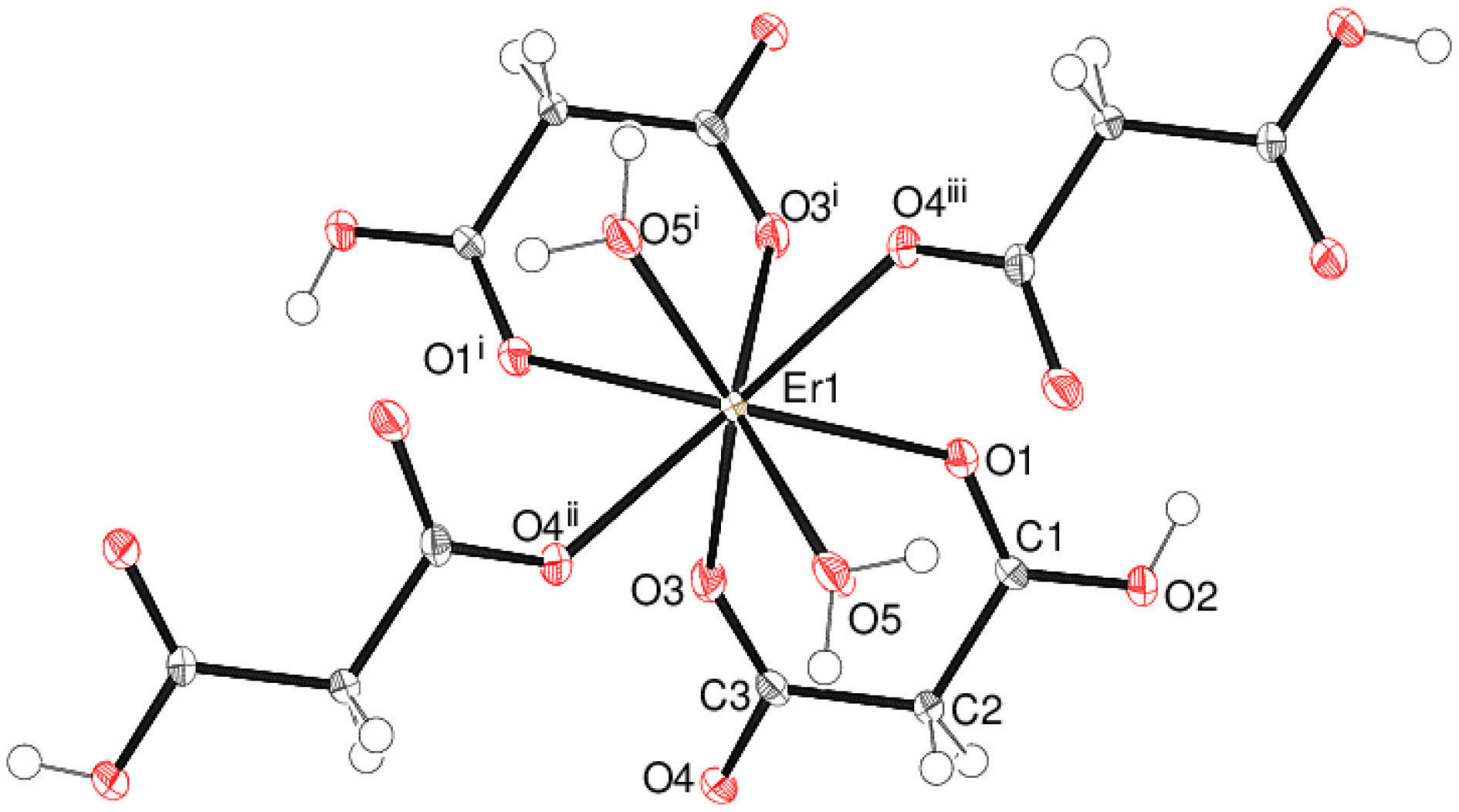
The asymmetric unit of **4** (50% displacement ellipsoids) expanded to show the full metal coordination sphere.

**Figure 6:**
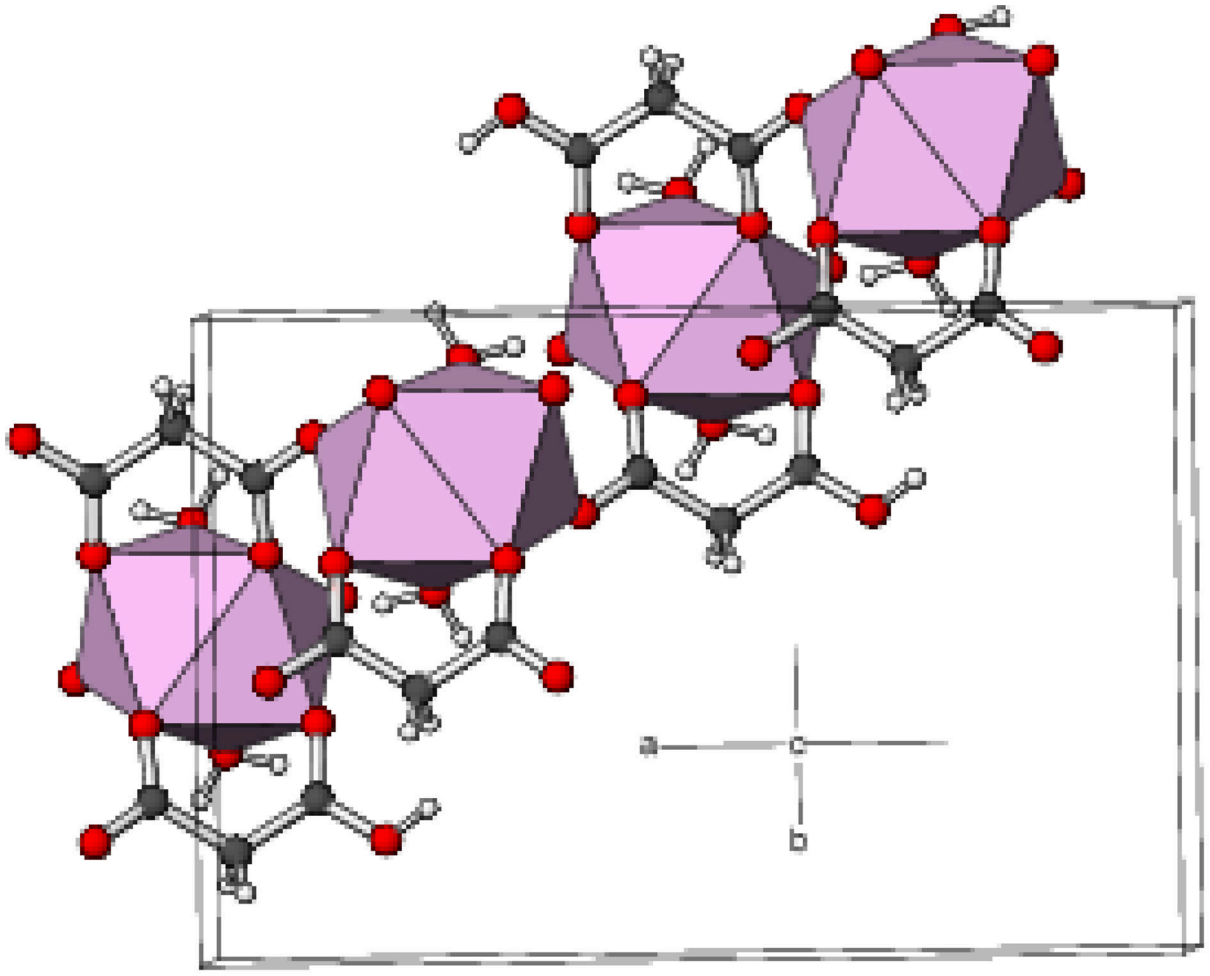
Part of a (110) chain in the structure of **4** with the ErO_8_ moieties represented as pink polyhedra and C, H, and O atoms shown as dark grey, white, and red spheres, respectively.

Complex **4** crystallizes in the unusual orthorhombic space group *Fdd*2. The asymmetric unit of **4** depicted in Figure 5 contains one erbium(III) ion (site symmetry 2), a malonate ligand, a hydrogen malonate anion and two water molecules. To balance the charge of Er^3+^, the malonate species must represent statistically disordered malonate (C_3_H_2_${\text{O}}_{4}^{2-}$) and hydrogen malonate (C_2_H_3_${\text{O}}_{4}^{-}$) ions, with half of the O2 oxygen atoms in the crystal bearing a proton. The coordination polyhedra of Er^3+^ ion is a square antiprism defined by two chelating ligands (bonding *via* O1 and O3), two monodentate malonate ligands (*via* O4) and two water molecules. In both square faces (O1/O1^i^/O3/O3^i^ and O4^ii^/O4^iii^/O5/O5^i^), the water molecules are located at “*trans”* positions (Figure S10). The dihedral angle between these planes is 0° (by symmetry) and the metal ion is displaced from them by −1.303(2) and 1.186(2) Å, respectively. The average Er–O bond length is 2.334(4) Å (Table S3) that agrees well with the reported values (Delgado et al., 2016). The chelate conformation is an asymmetric boat, with C2 and Er1 deviating from C1/O1/C3/O3 (r.m.s. deviation = 0.001 Å) by 0.324(6) and 0.827(6) Å, respectively. The C3–O3 and C3–O4 bond lengths in the ligand are almost the same, indicating the delocalization of charge. However, C1–O2 is much longer than C1–O1 as a result of its disordered protonation, as described above. It is notable that O2 (the protonated O atom) does not coordinate to the metal ion. The coordination mode of the C1/O1/O2 carboxyl group is η^1^ (monodentate) and that of C3/O3/O4 is μ_2_-η^1^:η^1^ (bridging). In the extended structure of **4** the malonate ligands are bound in chelating form on one side (through O1 and O3) and as a bridging entity (*via* O4) from the other side (μ_2_-κ^2^O,O'κO” coordination mode). The repeating units are cross-linked to generate a dense two-dimensional network, with no evidence of channels or pores. A part of the chain propagating in the (110) direction is expressed in Figure 6.

The structure of the compound does not match with any of the earlier reported structures of Ln^3+^complexes involving the hydrogen malonate ligand (Wenmei et al., 1992; Marrot and Trombe, 1994; Delgado et al., 2016).

**Figure 7:**
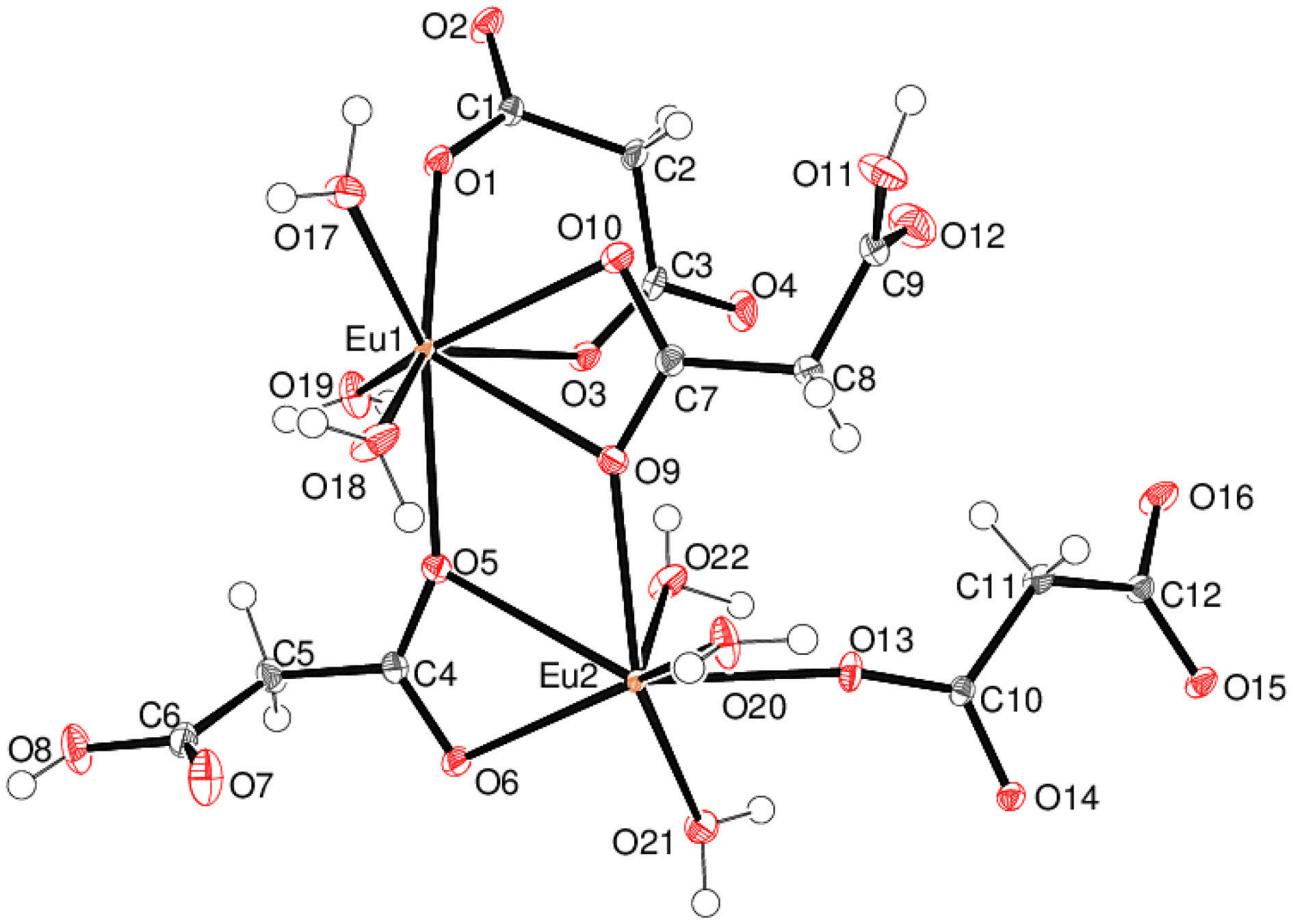
The asymmetric unit of **5** (50% displacement ellipsoids) with the non-coordinated water molecules of crystallization omitted for clarity.

Complex **5** is a 2D polymer consisting of neutral dinuclear asymmetric units (Figure 7) composed of two Eu^3+^ ions, two malonate dianions, two hydrogen malonate ions, six coordinated water molecules, and four water molecules of crystallization (one of which is disordered over two adjacent sites). In each dinuclear unit the two europium centers (Eu1 and Eu2) are bridged by the carboxylate groups of two hydrogen malonate ions to form a four membered Eu-O-Eu-O ring. Both metal ions are nine-coordinated by six carboxylate and three water oxygen atoms. The increase in coordination number from eight (for Gd^3+^ - Er^3+^) to nine (for Eu^3+^) is due to its larger size as compared with the others (Bünzli, 2014). The coordination polyhedron around Eu1 can be described as a distorted capped square antiprism (Figure S11) with O1/O3/O4^i^/O5 and O9/O10/O17/O18 sets (two water molecules *cis*) forming the square faces and O19 (water molecule) as the cap. The dihedral angle between the squares is 2.8(1)° and Eu1 is displaced from them by 0.7244(12) and −1.6269(12) Å, respectively. Eu2 possesses a similar coordination polyhedron, the squares of which are defined by O9/O13/O14^ii^/O15^ii^ and O5/O6/O21/O22 (water molecules *cis*) [dihedral angle between them = 4.61(9)°] and the cap by O20. Eu2 is displaced from these squares by −0.7270(11) and 1.6413(12) Å, respectively. The malonate dianions behave as bridging as well as chelating (forming a six-membered chelating ring through O1 and O3) ligands adopting μ_2_-κ^2^O,O'κO” coordination mode. The hydrogen malonate ions bind only on one side in a μ_2_-κO,κ^2^O' manner. The carboxylate bonding modes in 5 are more varied than in 1–4: C1/O1/O2 is monodentate to Eu1 (η1 mode); C3/O3/O4 is bridging to two Eu1 atoms (μ_2_-η^1^:η^1^ mode); C4/O5/O6 bonds to both Eu1 and Eu2 in chelating-bridging μ_2_-η^2^:η^1^ mode; C7/O9/O10 is chelating-bridging (μ_2_-η^2^:η^1^ mode); C6/O7/O8 and C9/O11/O12 are protonated at O8 and O12, respectively and these two groups do not coordinate to the metal ion; C10/O13/O14 is bridging (μ_2_-η^1^:η^1^); C12/O15/O16 is monodentate (η^1^ mode). The ligands have different conformations as indicated by the following dihedral angles between the carboxylate groups: C1/O1/O2 + C3/O3/O4 = 34.4(3)°; C4/O5/O6 + C6/O7/O8 = 72.9(3)°; C7/O9/O10 + C9/O11/O12 = 87.0(3)°; C10/O13/O14 + C12/O15/O16 = 28.5(3)°.

**Figure 8:**
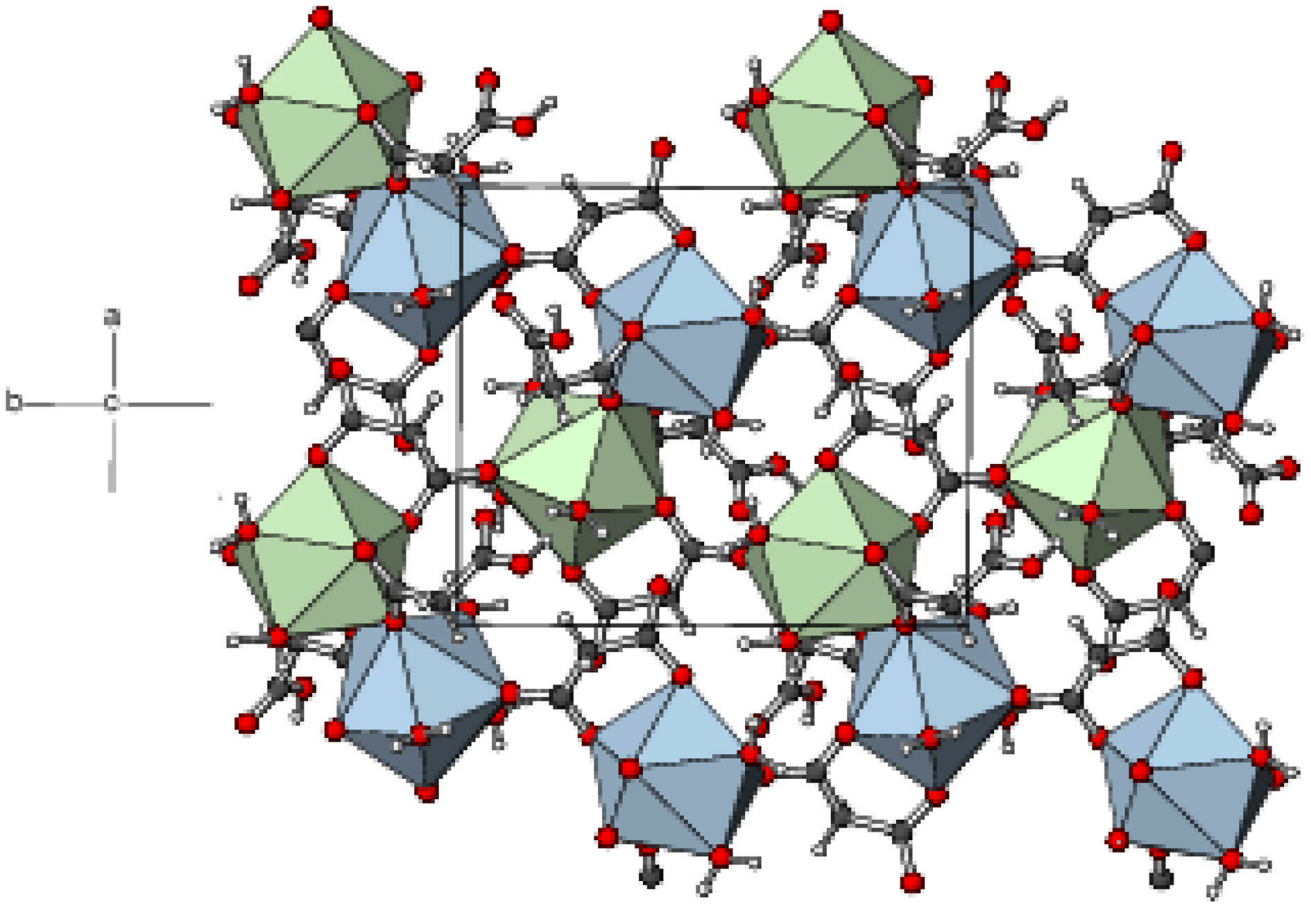
Part of a (001) layer in the structure of **5** with the Eu1O_9_ and Eu2O_9_ moieties represented as blue and green polyhedra, respectively and C, H, and O atoms shown as dark grey, white, and red spheres, respectively.

The mean Eu–O distances are 2.454(2) and 2.457(2) Å for Eu1 and Eu2, respectively (Table S4), which are comparable to the literature data (Hansson, 1973a; Hernández-Molina et al., 2002; Zhang et al., 2007; Jin et al., 2012). For both metal ions, the charge-assisted Eu–O bonds from the deprotonated carboxylate groups tend to be the shortest. In the extended structure of **5**, the europium polyhedra share an edge, *via* O5 and O9, resulting in a Eu1⋯ Eu2 separation of 4.3486 (4) Å. The ligands bridge the dinuclear species into (001) 2D sheets (Figure 8). Numerous O–H⋯ O hydrogen bonds (Table S9) consolidate the packing: the bonds from the O8 and O11 carboxyl groups (one to another ligand O atom and one to a water molecule) have notably shorter H⋯ O separations than the bonds arising from the water molecules.

### Comparative Structural Analysis

The crystal structures of several lanthanide compounds containing malonate ligand have been reported, which describe the ability of the malonate ion to adopt monodentate, chelating, and bridging coordination modes. The six-membered chelate ring through the two carboxylate groups is quite common in the lanthanide complexes (Delgado et al., 2016). In the present series of complexes, the malonate^2−^ ligand adopts two types of coordination modes; μ_3_-κ^2^O,O’κO”,κO”’ (**1**–**3**) and μ_2_-κ^2^O,O’κO” (**4**,**5**) leading to 2D network structures.

A closer look at the previously reported structures of the Ln(III)-malonate (mal^2−^) compounds shows that most of them are dinuclear having the general formula, [Ln_2_(mal)_3_(H_2_O)_6_]_*n*_^.^xH_2_O (Delgado et al., 2016), (Ce, Sm) (Doreswamy et al., 2003; Chrysomallidou et al., 2010) (Pr), (Hansson, 1973b; Doreswamy et al., 2005), (Nd) (Hernández-Molina et al., 2002; Zhang et al., 2007), (Eu) (Hernández-Molina et al., 2003; Cañadillas-Delgado et al., 2006) (Gd), (Fang et al., 2008), (Dy), or [Ln_2_(mal)_3_(H_2_O)_5_]_*n*_·xH_2_O (Delgado et al., 2016) (Ho, Tb, Dy, Er, Yb) (Hernández-Molina et al., 2003; Cañadillas-Delgado et al., 2006) (Gd) (Hansson, 1973a), (Eu). Some of them exist in the mononuclear form (Wenmei et al., 1992; Marrot and Trombe, 1994; Chrysomallidou et al., 2010; Silva et al., 2010). The trivalent lanthanide cations (because of their high charge and small size) have high affinity for water and therefore, the coordination sphere is usually completed by water molecules. Most of the structures are assembled into 3D coordination frameworks, while some as 1D or 2D polymers. The richness of structural architectures could be related to the versatility of malonate coordination modes and high values of the coordination number and flexibility of geometries of the Ln(III) ions.

In the majority of the complexes, the metal ions exhibit the coordination number nine, and the common geometries are tri-capped trigonal prism and distorted monocapped square antiprism (Delgado et al., 2016). In [Pr_2_(mal)_3_(H_2_O)_6_] and its dihydrate, the coordination environment is distorted capped tetragonal antiprism (Chrysomallidou et al., 2010). [Gd_2_(mal)_3_(H_2_O)_5_]_*n*_^.^2*n*H_2_O is eight-coordinated in which the Gd environment is distorted square antiprismatic (Cañadillas-Delgado et al., 2006). Complexes with coordination number of ten are also known. For example, in 1D polymeric [CeCl(mal)(H_2_O)_3_]^.^0.5(H_2_O), the cerium center adopts a highly distorted dodecahedron (Silva et al., 2010). The coordination polyhedra of metal ions in [La(H_2_O)_2_(mal)(mal-H)]^.^H_2_O and [PrCl(mal)(H_2_O)_3_]_*n*_·0.5*n*H_2_O are best described as distorted tetracapped trigonal prism (Marrot and Trombe, 1994) and bicapped tetragonal antiprism (Chrysomallidou et al., 2010), respectively. The extensive network of hydrogen bonds in the polymers enhances their structural stability.

The structures of the complexes presented here (**1**–**5**) do not possess similarity to any of the previously reported structures. Four of the five complexes exist as mononuclear 2D polymers, while the fifth (**5**) is dinuclear. Two complexes (**4** and **5**) are non-ionic as usual, but three (**1**–**3**) contain nitrate as a counter ion, which are so far unprecedented. In complexes **1**–**4**, the metal ions are eight-coordinated. The increase in coordination number from eight (for Gd^3+^-Er^3+^) to nine (for Eu^3+^) is due to its larger size compared with the others. The change in coordination number from nine to eight can be related to the lanthanide contraction (Bünzli, 2014).

### Photoluminescence Properties

**Figure 9:**
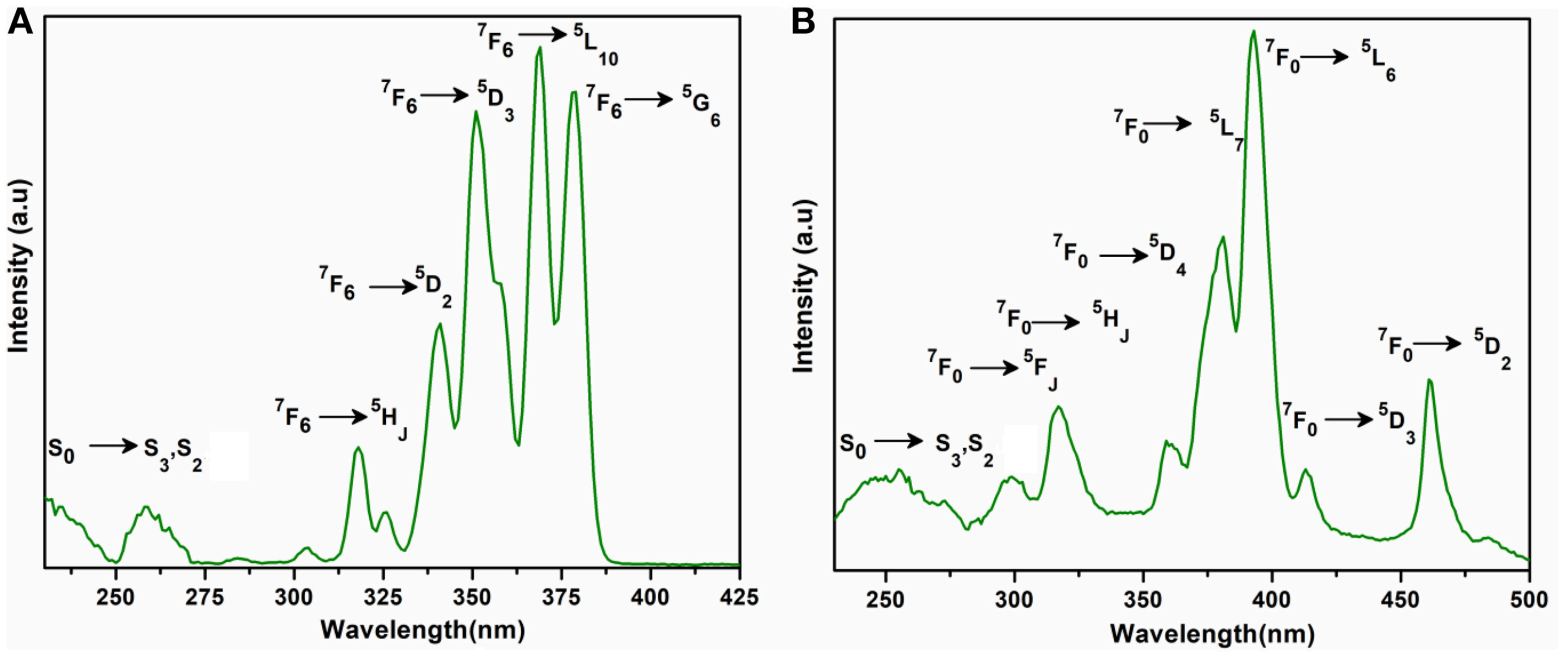
Solid state excitation spectra of **2 (A)** and **5 (B)** to monitor emission wavelength at 548 and 619 and respectively.

**Figure 10:**
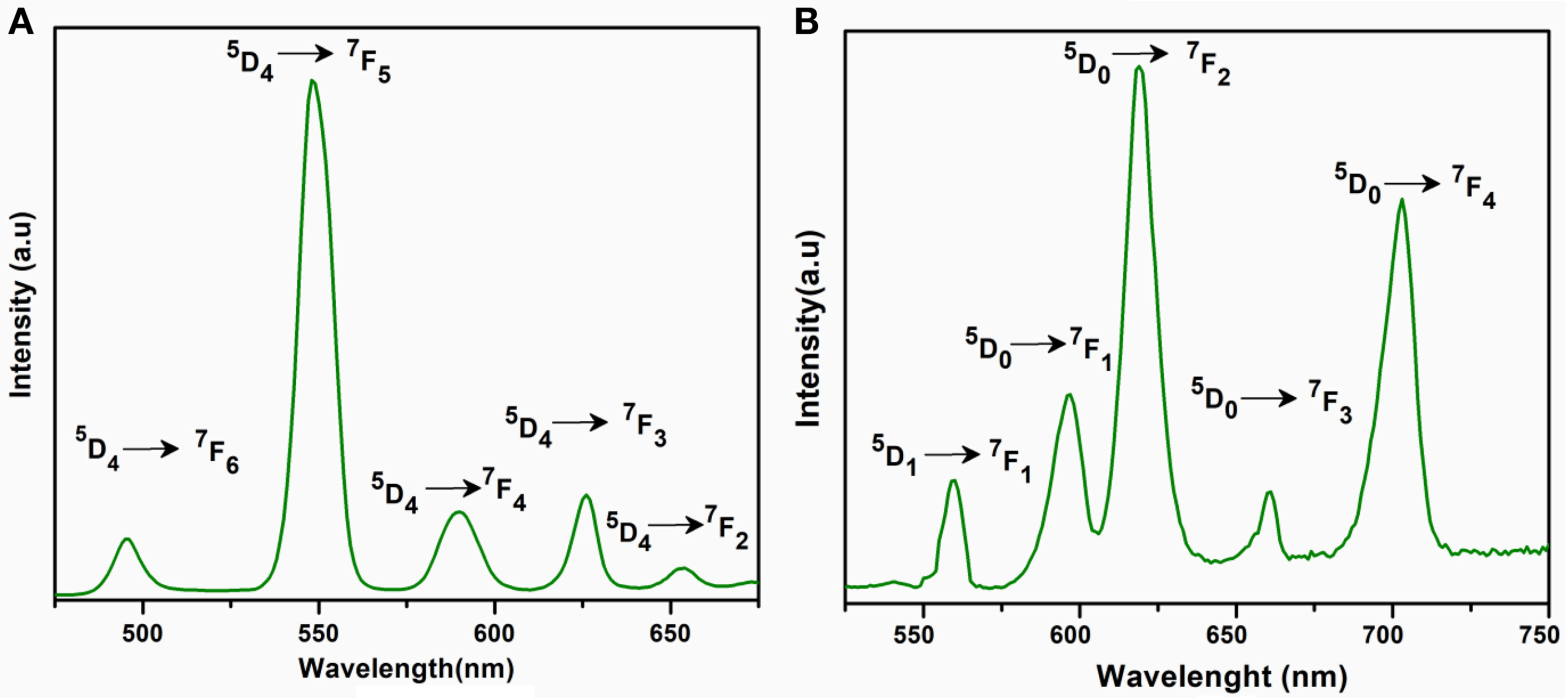
Solid state emission spectra of **2 (A)** and **5 (B)** excited at 365 and 381 nm respectively.

The excitation and emission spectra of **1–5** were recorded in the solid state at room temperature. All these compounds **1–5** exhibited luminescence in the visible region but most excellent results were obtained for compounds **2** and **5**. The excitation spectrum of **2** was recorded to monitor the strongest emission of terbium (^5^D_4_ → ^7^F_5_) at 548 nm. The spectrum of **2** (Figure 9A) exhibited ligand excited peaks between 230 and 270 nm which correspond to the S_0_ → S_3_, S_2_ of the ligand transitions. The peaks in the terbium spectrum were observed at 318, 341, 351, 365, and 379 nm which correspond to the ^7^F_6_ → ^5^H_7_, ^5^D_0, 1_, ^7^F_6_ → ^5^D_2_, ^7^F_6_ → ^5^D_3_, ^7^F_6_ → ^5^L_10_, and ^7^F_6_ → ^5^G_6_ transitions, respectively. The excitation spectrum of **5** was recorded to monitor the strongest emission of europium (^5^D_0_ → ^7^F_2_) at 619 nm. The spectrum of **5** (Figure 9B) also exhibited broad band between 230 and 275 nm that represents the ligand excitations S_0_ → S_3_ and S_0_ → S_2_. The excitation peaks of europium are located at 300, 317, 360, 381, 393, 413, and 461 nm attributed to the ^7^F_0_ → ^5^F_J_, ^7^F_0_ → ^5^H_J_, ^7^F_0_ → ^5^D_4_, ^7^F_0_ → ^5^L_7_, ^7^F_0_ → ^5^L_6_, ^7^F_0_ → ^5^D_3_, and ^7^F_0_ → ^5^D_3_ transitions, respectively. The major ligand excitation signal S_0_ → S_1_ expected at 370 nm might be superimposed on strong peaks ^7^F_6_ → ^5^L_10_ and ^7^F_0_ → ^5^L_7_ in **2** and **5**, respectively. The excitation spectra of **2** and **5** depict that the ligand absorbs at shorter wavelength (230-−280 nm) for excitation which helps to sensitize the symmetry forbidden *f-f* transition of Tb(III) and Eu(III) ions, respectively. The emission spectrum of **2** under the excitation wavelength of 365 nm is shown in Figure 10A which exhibits five characteristic *f–f* emission peaks of terbium at 496, 548, 584, 626, and 654 nm attributed to the ^5^D_4_ → ^7^F_6_, ^5^D_4_ → ^7^F_5_, ^5^D_4_ → ^7^F_4_, ^5^D_4_ → 7F_3_, and ^5^D_4_ → 7F_2_ transitions, respectively. The strongest emission is observed by electric dipole transition at 548 nm (^5^D_4_ → ^7^F_5_) due to the induced effect of the coordinated ligand, which confers to the intense green luminescence output from the solid sample when irritated under UV light (Hou et al., 2013; Bogale et al., 2017a,b). In some previously reported terbium carboxylate polymers, the intensity of this emission is quenched in the presence of Fe^3+^ and nitroaromatics, and can be used as a sensor for the detection of metal ions and nitro compounds (Bogale et al., 2017a,b). The magnetic transition at 490 nm (^5^D_4_ → ^7^F_6_) is relatively very weak with intensity ratio 1:6 (^5^D_4_ → ^7^F_6_: ^5^D_4_ → ^7^F_5_) that is almost insensitive to the coordinated environments (Bogale et al., 2017a,b). These peaks have been observed in other terbium complexes (Zhao et al., 2004; Hou et al., 2013, 2014). The emission spectrum of **5** under the excitation of 381 nm is shown in Figure 10B which exhibits five characteristic *f–f* emission peaks of europium 560 nm (^5^D_1_ → ^7^F_1_), 597nm (^5^D_0_ → ^7^F_1_), 619 nm (^5^D_0_ → ^7^F_2_), 661 nm (^5^D_0_ → ^7^F_3_), and 560 nm (^5^D_0_ → ^7^F_4_). Among them an induced electric dipole transition ^5^D_0_ → ^7^F_2_ is the most intense which is hypersensitive to the chemical environment in the vicinity of Eu(III) ion and responsible for the strong red emission when irradiated under UV light (Hou et al., 2014). The magnetic dipole transition at 597 nm (^5^D_0_ → ^7^F_1_) that is less sensitive to the coordinated environment is relatively weak compared with the electric dipole transition. The Eu(III) transition rule states that the magnetic dipole transition (^5^D_0_ → ^7^F_1_) will be more intense when there exists a center of inversion. The intensity of electric dipole transition ^5^D_0_ → ^7^F_2_ transition will be decreased as the site symmetry of Eu(III) ion increases (Hou et al., 2014). By comparing the intensity of ^5^D_0_ → ^7^F_2_ (619 nm) and ^5^D_0_ → ^7^F_1_(597nm) for **5**, the intensity ratio is about 4:1 which suggests that europium ion exists in the low symmetry environment and there is no center of inversion as revealed by the X-ray structural analysis of compound **5** (Gu and Xue, 2006). The excitation and emission spectra of **3** (Figure S12) exhibited sharp peaks with broad bases at 370 (^5^I_8_ → ^3^G_6_) and 570 nm (^5^S_2_, ^5^F_4_ → ^5^I_8._), respectively. In addition, few broad peaks are observed between 400 and 500 nm in the emission spectrum of **3** which are difficult to designate. The emission spectra of **1** (Figure S13B) and **4** (Figure S14B) are different from **2**, **3**, and **5**. No characteristic peaks of gadolinium and erbium are observed in the emission spectra of **1** and **4** except broad emission bands at 560 and 470 nm, respectively.

Plush and Gunnlaugsson reported that a dinuclear europium(III) bismacrocyclic complex (Plush and Gunnlaugsson, 2007) showed significant enhancement in europium(III) emission when titrated with malonic acid at pH 6.5. When this dinuclear complex was titrated with other dicarboxylates such acetate, succinate, aspartate, and glutarate acids at the same pH, the Eu(III) emission on all occasions was quenched (Plush and Gunnlaugsson, 2007). Thus, we believe that malonic acid is most suitable to produce excited states that help to sensitize the lanthanide luminescence compared with the other aliphatic dicarboxylates.

### Magnetic Properties

The room temperature magnetic moments of complexes **1–5** are provided in Table 3. The experimental μ_eff_ values for these complexes are near to the Hund (except for 5) and Van Vleck (Van Vleck, 1932) values for the applicable free Ln(III) ion. The non-zero moment for **5** can be ascribed to the contribution of low-lying excited states to the magnetism, as observed in other europium(III) complexes (Ferenc et al., 2013a,b). These data indicate that the energies of the 4*f* orbitals in the title compounds differ a little from the free lanthanide(III) ions due to the fact that 5*s*^2^ and 5*p*^6^ orbitals are completely filled and strongly shield the 4*f* orbitals. Thus, it supports the assumption of the electrostatic nature of metal-ligand bonding in these compounds (Sinha, 2012; Kettle, 2013).

**Table 3:** The room temperature experimental values of effective magnetic moment (μ_eff_) for **1-5** and Van Vleck and Hund magnetic moment (μ_eff_) for free lanthanide ions.

**Complex**	**Trivalent ion**	**Configuration 4*f^***n***^***	**μ(μB) (Van Vleck)**	**μ(μB) (Hund)**	**μ_**eff**_(μB) Experimental**
**1**	Gd	4*f*^7^	7.94	7.94	8.38
**2**	Tb	4*f*^8^	9.70	9.70	9.82
**3**	Ho	4*f*^10^	10.60	10.60	9.95
**4**	Er	4*f*^11^	9.60	9.60	9.53
**5**	Eu	4*f*^6^	3.40–351	0.00	3.98

μB = Bohr magneton

### Computational Study of Optoelectronic Properties of [Gd(C_3_H_2_O_4_)(H_2_O)_4_]_n_·NO_3_}_n_ (1)

The optoelectronic properties of compound **1** were explored using first principle methods (Supporting Information). The theoretical calculations have been performed within periodic boundary conditions (PBC) using DFT functional called PBE (in generalized gradient approximation) in solid-state calculations. Further details about computational parameters including Brillouin Zone (BZ) sampling and energy cut-offs are given in the Supporting Information. All the computational calculations were performed using Cambridge Serial Total Energy Package (CASTEP) in Material Studio (Carter et al., 2006). A detailed comparison of optimized geometry and experimental single crystal geometry of compound **1** is presented in Table S10 and Figure S16, which indicates the reliability of our chosen geometry in the present investigation.

### Density of States (DOS)

**Figure 11:**
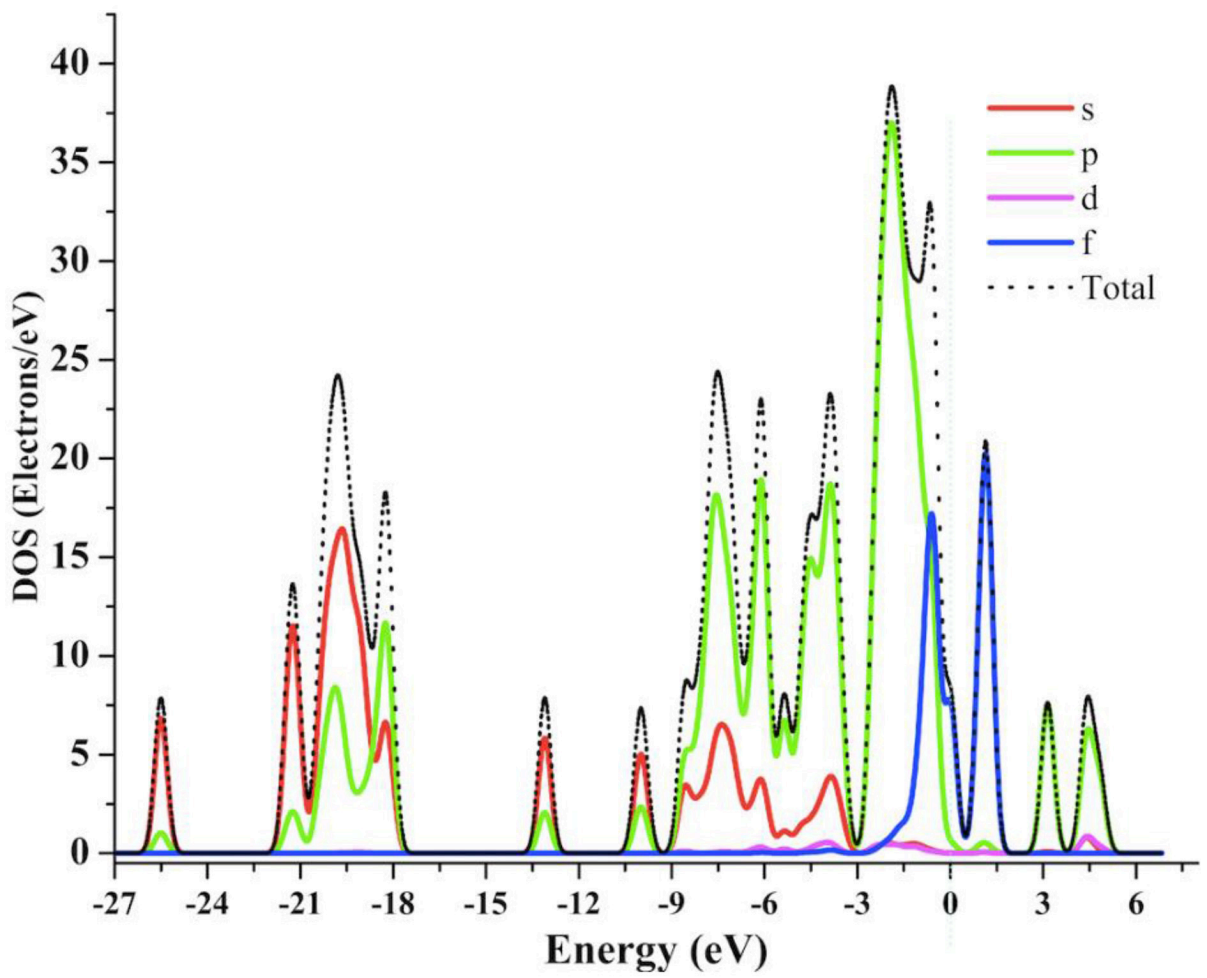
The calculated TDOS and PDOS graph for **1** at GGA-PBE level of theory.

In order to understand the structure-property relationship, we have calculated total density of states (TDOS) and partial density of states (PDOS) for compound 1. The TDOS and PDOS projected into individual states contributions of the molecule were calculated for compound 1 as shown in Figure 11. The total DOS shows a lot of structural patterns that can be better understood by looking at the PDOS. It can be seen that in deep valence band the contributions of *s*- and *p*-states from oxygen atoms are significant in the range of −25 to −3 eV, which indicates significant ligand contributions in the molecular structure. On the other hand, around the Fermi-level the contributions from *p*- and *f*-states are significant. Hybridization effects can also be analyzed using PDOSs from Figure 10. The significant contributions of *p*-states of O atoms and *f*-states of Gd atoms around −0.5 eV indicates the strong bonding character or energy states hybridization of gadolinium atoms with oxygen atoms of ligands.

### Optical Properties

**Figure 12:**
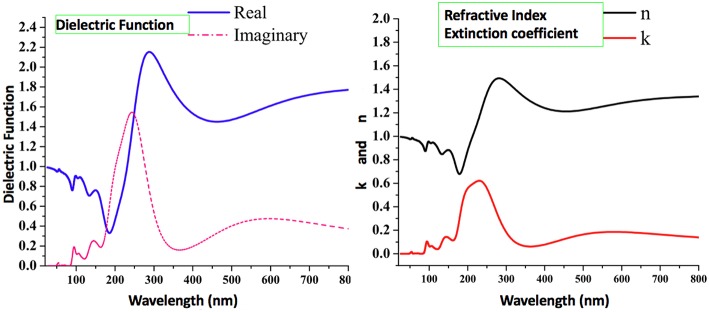
The calculated optical properties of **1**, including real and imaginary parts of the dielectric function, refractive index, and extinction.

In the past, the photophysical and electroluminescence properties of rare earth metals have been studied very keenly because of their many technological applications i.e., optical amplifiers, luminescent solar concentrators, and active waveguides (Kenyon, 2002; Kido and Okamoto, 2002; Armelao et al., 2010; Katkova and Bochkarev, 2010; Heffern et al., 2013; Reisfeld, 2015). Along similar lines, we have calculated some important optical parameters, including real and imaginary parts of dielectric function, refractive index, extinction coefficient and absorption spectrum in the solid-state for **1** using periodic boundary conditions. The real and imaginary parts of the dielectric function are presented in Figure 12. The dielectric function is the response of the material to the alternating electric field. Experimentally, dielectric function is calculated using dielectric techniques e.g., dielectric relaxation spectroscopy (DRS). In the present investigation, the optical functions of compound (**1**) are calculated for photon wavelength up to 800 nm to the direction of polarization vector [100]. For compound **1**, its real and imaginary parts of the dielectric function show peaks of maximum dielectric function values (real = 2.2 and imaginary = 1.5) in the range of 200 nm to 300 nm, which indicates that the maximum interaction of light occurs in this range. The refractive index and extinction coefficients also show the highest values of *n* (1.52) and *k* (0.64) at similar wavelengths as that of dielectric function in the UV region. These values indicate by how much the value is bent when entering the material.

**Figure 13:**
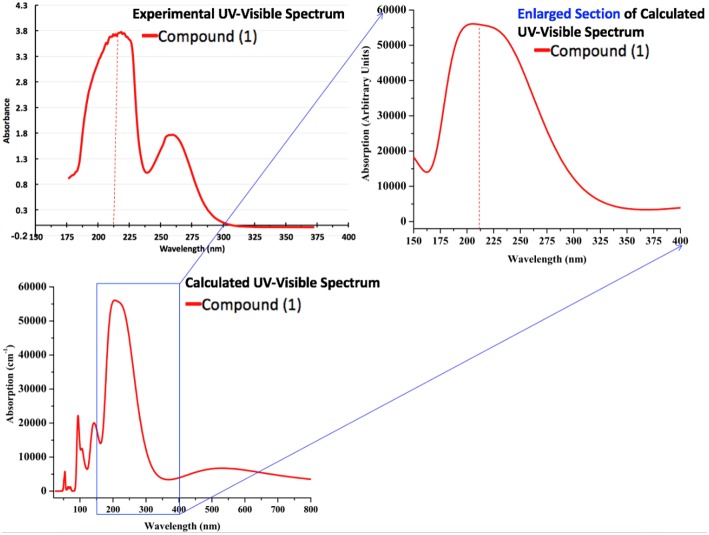
The calculated (PBE-GGA) and experimental (in ethanol) absorption spectra of compound **1**.

Moreover, we have also calculated the UV-Visible spectrum for compound **1** by using the PBE-GGA method for solid-state crystal structure. In addition to the commutated UV-Visible spectrum, we have also experimentally recorded the UV-Visible spectrum in ethanol solution. Both the calculated and experimental spectra are illustrated in Figure 13. It is important to mention that the experimental spectrum is only available in the range of 190–400 nm due to the limits of our spectrophotometer. Owing to the above situation and to make a comprehensive comparison of computed and experimental UV-Visible spectra, we have also presented the enlarged part of the computationally generated UV-Visible spectrum in the range of 150–400 nm as shown in Figure 13. A comparison of computationally generated and experimental UV-Visible spectra shows that they are in reasonable agreement with each other. The maximum absorption wavelengths in both spectra are found to be around ~215 nm. Moreover, the absorption ranges for both the spectra are between 175 and 300 nm. The experimental spectrum for compound **1** shows one shoulder peak which might be due to the fact that it has been measured in ethanol while the computationally generated spectrum is in the solid state. The absorption values display maximum amplitudes in the range of 200–250 nm with good absorption coefficients in the ultraviolet region that could be valuable for the possible application of compound **1** as a UV sensor. Thus, we believe that these optical properties can be very helpful to offer a theoretical basis for the experimental analysis of photophysical properties in future, and to get new physical insights from the molecules to materials.

## Conclusions

Five new lanthanide-malonate coordination polymers (1–5) with two- or three-dimensional connectivity have been synthesized and their crystal structures were determined by X-ray crystallography. The complexes **1**–**4** are monoculear and the central Ln^3+^ ions are eight-coordinate assuming square antiprism geometry. Complex **5** is dinuclear with the nine-coordinated Eu^3+^ ions possessing the mono-capped square antiprism polyhedra. The malonate and hydrogen malonate ligands show typical versatility in their chelating, bridging, and combined bonding modes to the different metal ions. So far as we are aware, all three are new crystal structure types. Most of the known compounds such as, [Gd_2_(C_3_H_2_O_4_)_3_(H_2_O)_6_]*n*·2*n*H_2_O (Cañadillas-Delgado et al., 2006) [Er_2_(C_3_H_2_O_4_)_3_(H_2_O)_5_]*n*·2*n*H_2_O, (Delgado et al., 2016), and [Eu_2_(C_3_H_2_O_4_)_3_(H_2_O)_6_]*n*·2*n*H_2_O (Hernández-Molina et al., 2002) contain dianionic malonate ligands, while only a few reports are available on the hydrogen malonate complexes (Wenmei et al., 1992; Marrot and Trombe, 1994). The IR spectra also indicate the presence of hydrogen malonate ligand in **4** and **5**. The results of the thermogravimetric analysis verified the composition of the investigated complexes. The photoluminescence spectra of **2** and **5** exhibit characteristic emission of Tb(III) and Eu(III), respectively. Additionally, we have used the first principle calculations for compound **1** to explore its optoelectronic properties. The prediction of dielectric function, refractive index, extinction coefficient and absorption spectrum shed light on prospective applications as optical materials. The maximum dielectric function and other absorption coefficients in the UV region for compound **1** indicate its potential application as a UV sensor. A comparison of computationally generated and experimental UV-Visible spectra shows that they are in reasonable agreement with each other having maximum absorption around ~215 nm. Thus, we strongly believe that the present set of synthesis, characterization, and computational insights will evoke the significant interests of scientific community in the chemistry of rare-earth coordination polymers.

## Author Contributions

XC, SA, and IK devised the project, the main conceptual ideas, and proof outline. SH synthesized the compounds, performed analyses and wrote the manuscript. SM carried out the computational and theoretical work. ME collected the crystal date and helped in structure solutions. WH refined and drew images of the structures and wrote the crystal description.

### Conflict of Interest Statement

The authors declare that the research was conducted in the absence of any commercial or financial relationships that could be construed as a potential conflict of interest.

## References

[B1] Adamo C. Maldivi P. (1997). Ionic versus covalent character in lanthanide complexes. A hybrid density functional study. Chem. Phys. Lett. 268, 61–68. 10.1016/S0009-2614(97)00177-2

[B2] Alhoshani A. Seliman A. A. Altoum A. O. Abuelizz H. A. Ahmad S. Altaf M. (2019). Synthesis, X-ray structure and *in vitro* cytotoxicity of trans-diammineplatinum(II) complexes of selenones, trans-[Pt (NH_3_)_2_ (selenone)_2_](NO_3_)_2_. Polyhedron 158, 234–240. 10.1016/j.poly.2018.09.010

[B3] Armelao L. Quici S. Barigelletti F. Accorsi G. Bottaro G. Cavazzini M. (2010). Design of luminescent lanthanide complexes: from molecules to highly efficient photo-emitting materials. Coord. Chem. Rev. 254, 487–505. 10.1016/j.ccr.2009.07.025

[B4] Benmerad B. Guehria-Laidoudi A. Bernardinelli G. Balegroune F. (2000). Polymeric tetraaquatris (malonato) dilanthanum(III) monohydrate. Acta. Crystallogr. C Cryst. Struct. Chem. 56, 321–323. 10.1107/S0108270199016182 10777935

[B5] Bogale R. F. Chen Y. Ye J. Yang Y. Rauf A. Duan L. (2017a). Highly selective and sensitive detection of 4-nitrophenol and Fe^3+^ ion based on a luminescent layered terbium(III) coordination polymer. Sens. Actuators B. Chem. 245, 171–178. 10.1016/j.snb.2017.01.177

[B6] Bogale R. F. Chen Y. Ye J. Zhang S. Li Y. Liu X. (2017b). A terbium(III)-based coordination polymer for selective and sensitive sensing of nitroaromatics and ferric ion: synthesis, crystal structure and photoluminescence properties. New J. Chem. 41, 12713–12720. 10.1039/C7NJ02492D

[B7] Buenzli J. C. G. (2015). On the design of highly luminescent lanthanide complexes. Coord. Chem. Rev. 293, 19–47. 10.1016/j.ccr.2014.10.013

[B8] Bünzli J. C. G. (2010). Lanthanide luminescence for biomedical analyses and imaging. Chem. Rev. 2110, 2729–2755. 10.1021/cr900362e 20151630

[B9] Bünzli J. C. G. (2014). Lanthanide coordination chemistry: from old concepts to coordination polymers. J. Coord. Chem. 67, 3706–3733. 10.1080/00958972.2014.957201

[B10] Calahorro A. J. Fairen-Jiménez D. Salinas-Castillo A. López-Viseras M. E. Rodríguez-Diéguez A. (2013). Novel 3D lanthanum oxalate metal-organic-framework: Synthetic, structural, luminescence and adsorption properties. Polyhedron 52, 315–320. 10.1016/j.poly.2012.09.018 23286441

[B11] Cañadillas-Delgado L. Pasan J. Fabelo O. Hernandez-Molina M. Lloret F. Julve M.. (2006). Two-and three-dimensional networks of gadolinium(III) with dicarboxylate ligands: synthesis, crystal structure, and magnetic properties. Inorg. Chem. 45, 10585–10594. 10.1021/ic061173d 17173413

[B12] Caravan P. Ellison J. J. Mcmurry T. J. Lauffer R. B. (1999). Gadolinium(III) chelates as MRI contrast agents: structure, dynamics, and applications. Chem. Rev. 99, 2293–2352. 10.1021/cr980440x 11749483

[B13] Carter R. Sloan J. Kirkland A. I. Meyer R. R. Lindan P. J. Lin G.. (2006). Correlation of structural and electronic properties in a new low-dimensional form of mercury telluride. Phys. Rev. Lett. 96:215501. 10.1103/PhysRevLett.96.215501 16803245

[B14] Chrysomallidou K. E. Perlepes S. P. Terzis A. Raptopoulou C. P. (2010). Synthesis, crystal structures and spectroscopic studies of praseodymium(III) malonate complexes. Polyhedron 29, 3118–3124. 10.1016/j.poly.2010.08.020

[B15] Cui G.-H. Li J.-R. Zhang R.-H. Bu X.-H. (2005). Hydrothermal synthesis, crystal structures and luminescent properties of two new Ln(III)–succinate (Ln = Eu, Tb) complexes exhibiting three dimensional networks. J. Mol. Struct. 740, 187–191. 10.1016/j.molstruc.2005.01.049

[B16] Cundari T. R. Sommerer S. O. Strohecker L. A. Tippett L. (1995). Effective core potential studies of lanthanide complexes. J. Chem. Phys. 103, 7058–7063. 10.1063/1.470333

[B17] Daiguebonne C. Kerbellec N. Guillou O. Bünzli J. C. Gumy F. Catala L.. (2008). Structural and luminescent properties of micro-and nanosized particles of lanthanide terephthalate coordination polymers. Inorg. Chem. 47, 3700–3708. 10.1021/ic702325m 18366158

[B18] Deacon G. Phillips R. (1980). Relationships between the carbon-oxygen stretching frequencies of carboxylato complexes and the type of carboxylate coordination. Coord. Chem. Rev. 33, 227–250. 10.1016/S0010-8545(00)80455-5

[B19] Deacon G. B. Forsyth M. Junk P. C. Leary S. G. Moxey G. J. (2006). Synthesis and structural diversity of rare earth anthranilate complexes. Polyhedron 25, 379–386. 10.1016/j.poly.2005.07.005

[B20] Delgado F. S. Lorenzo-Luís P. Pasán J. Cañadillas-Delgado L. Fabelo O. Hernández-Molina M. (2016). Crystal growth and structural remarks on malonate-based lanthanide coordination polymers. Cryst. Eng. Comm. 18, 7831–7842. 10.1039/C6CE01360K

[B21] Dolg M. (2015). Computational Methods in Lanthanide and Actinide Chemistry. John Wiley & Sons 10.1002/9781118688304

[B22] Doreswamy B. Mahendra M. Sridhar M. Prasad J. S. Varughese P. George J. (2005). A novel three-dimensional polymeric structure of crystalline neodymium malonate hydrate. Mater. Lett. 59, 1206–1213. 10.1016/j.matlet.2004.12.029

[B23] Doreswamy B. Mahendra M. Sridhar M. Prasad J. S. Varughese P. Saban K. (2003). Structural studies on praseodymium malonate hydrate. J. Mol. Struct. 659, 81–88. 10.1016/j.molstruc.2003.08.001

[B24] Dos Santos C. M. Harte A. J. Quinn S. J. Gunnlaugsson T. (2008). Recent developments in the field of supramolecular lanthanide luminescent sensors and self-assemblies. Coord. Chem. Rev. 252, 2512–2527. 10.1016/j.ccr.2008.07.018

[B25] Earnshaw A. (2013). Introduction to Magnetochemistry. London; New York, NY: Elsevier; Academic Press.

[B26] Eisenstein O. Maron L. (2002). DFT studies of some structures and reactions of lanthanides complexes. J. Organomet. Chem. 647, 190–197. 10.1016/S0022-328X(01)01407-3

[B27] Fang Z.-Q. Zeng R.-H. Song Z.-F. Yang M. (2008). Poly [hexaaquatri-μ-malonato-didysprosium (III)]. Acta Crystallogr. Sect. E, Struct. Rep. Online 64, m877–m877. 10.1107/S1600536808015961 21202748 PMC2961881

[B28] Farrugia L. J. (2012). WinGX and ORTEP for Windows: an update. J. Appl. Cryst. 45, 849–854. 10.1107/S0021889812029111

[B29] Faulkner S. Pope S. J. Burton-Pye B. P. (2005). Lanthanide complexes for luminescence imaging applications. Appl. Spectrosc. Rev. 40, 1–31. 10.1081/ASR-200038308

[B30] Ferenc W. Cristóvão B. Sarzynski J. (2013a). Magnetic, thermal and spectroscopic properties of lanthanide (III) 2-(4-chlorophenoxy) acetates, Ln (C_8_H_6_ClO_3_)_3_∙*n*H_2_O. J. Serb. Chem. Soc. 78, 1335–1349. 10.2298/JSC121203043F

[B31] Ferenc W. Sadowski P. Cristovao B. Sarzynski J. (2013b). Investigation of some physicochemical properties of 4-nitrocinnamates of lanthanides (III). J. Chil. Chem. Soc. 58, 1753–1758. 10.4067/S0717-97072013000200025

[B32] Gao H.-L. Huang S.-X. Zhou X.-P. Liu Z. Cui J.-Z. (2018). Magnetic properties and structure of tetranuclear lanthanide complexes based on 8-hydroxylquinoline Schiff base derivative and β-diketone coligand. Dalton Trans. 47, 3503–3511. 10.1039/C8DT00063H 29431833

[B33] George T. Varughese S. Reddy M. (2016). Near-infrared luminescence of Nd 3+ and Yb 3+ complexes using a polyfluorinated pyrene-based β-diketonate ligand. RSC Adv. 6, 69509–69520. 10.1039/C6RA12220E

[B34] Gu X. Xue D. (2006). Selected controlled synthesis of three-dimensional 4d– 4f heterometallic coordination frameworks by lanthanide carboxylate subunits and silver centers. Cryst. Growth Des. 6, 2551–2557. 10.1021/cg060485o

[B35] Hansson E. (1973a). The crystal and molecular structure of penta-aquo Tris-malonato Di-Europiumum(III) Tri-hydrate. Acta. Chem. Scand. 27, 2827–2840. 10.3891/acta.chem.scand.27-2827

[B36] Hansson E. (1973b). Structural studies on the rare earth carboxylates. Acta. Chem. Scand. 27, 823–834. 10.3891/acta.chem.scand.27-0823

[B37] He H. Ma H. Sun D. Zhang L. Wang R. Sun D. (2013). Porous lanthanide–organic frameworks: control over interpenetration, gas adsorption, and catalyst properties. Cryst. Growth Des. 13, 3154–3161. 10.1021/cg400531j

[B38] Heffern M. C. Matosziuk L. M. Meade T. J. (2013). Lanthanide probes for bioresponsive imaging. Chem. Rev. 114, 4496–4539. 10.1021/cr400477t 24328202 PMC3999228

[B39] Hernández-Molina M. Lorenzo-Luis P. Ruiz-Pérez C. López T. Martín I. R. Anderson K. M. (2002). A phase transition in the novel three-dimensional compound [Eu_2_(mal)_3_ (H_2_O)_6_](H_2_mal = malonic acid). J. Chem. Soc., Dalton Trans. 3462–3470. 10.1039/B202649J

[B40] Hernández-Molina M. Lorenzo-Luis P. A. López T. Ruiz-Pérez C. Lloret F. Julve M. (2000). Generation of lanthanide coordination polymers with dicarboxylate ligands: synthesis, structure, thermal decomposition, and magnetic properties of the two-dimensional triaquatris (malonato) dipraseodymium(III) dihydrate {[Pr_2_(C_3_H_2_O_4_)_3_(H_2_O)_3_]·2H_2_O}. Cryst. Eng. Comm. 2, 169–173. 10.1039/B006256L

[B41] Hernández-Molina M. Ruiz-Pérez C. López T. Lloret F. Julve M. (2003). Ferromagnetic coupling in the three-dimensional malonato-bridged gadolinium (III) complex [Gd_2_(mal)_3_ (H_2_O)_6_](H_2_mal = Malonic Acid). Inorg. Chem. 42, 5456–5458. 10.1021/ic034175w 12950184

[B42] Hou Y. L. Cheng R. R. Xiong G. Cui J. Z. Zhao B. (2014). Structures, luminescent and magnetic properties of a series of (3, 6)-connected lanthanide–organic frameworks. Dalton Trans. 43, 1814–1820. 10.1039/C3DT52305E 24253449

[B43] Hou Y. L. Xiong G. Shen B. Zhao B. Chen Z. Cui J. Z. (2013). Structures, luminescent and magnetic properties of six lanthanide–organic frameworks: observation of slow magnetic relaxation behavior in the Dy III compound. Dalton Trans. 42, 3587–3596. 10.1039/c2dt32390g 23292215

[B44] Hussain S. Khan I. Akkurt M. Ahmad S. Tahir M. (2014). Synthesis and structural characterization of binuclear ytterbium (III) complexes with 2-amino and 3-amino benzoic acid. Russ. J. Coord. Chem. 40, 686–694. 10.1134/S107032841409005X

[B45] Hussain S. Khan I. Harrison W. T. Tahir M. (2015a). Crystal structures and characterization of two rare-earth-glutarate coordination networks: One-dimensional [Nd(C_5_H_6_O_4_)(H_2_O)_4_]·Cl and three-dimensional [Pr(C_5_H_6_O_4_)(C_5_H_7_O_4_)(H_2_O)]·H_2_O. J. Struct. Chem. 56, 934–941. 10.1134/S0022476615050169

[B46] Hussain S. Khan I. Harrison W. T. Tahir M. Ahmad S. (2015b). Crystal structures and characterization of two one-dimensional coordination polymers containing Ln^3+^ ions and anthranilate (C_7_H_6_${\text{NO}}_{2}^{-}$) anions. J. Struct. Chem. 56, 126–133. 10.1134/S0022476615010187

[B47] Hussain S. Khan I. U. Elsegood M. R. Jabeen N. Tahir M. N. Ahmad S. (2018). Synthesis and structural characterization of dinuclear cerium(III) and erbium (III) complexes of nicotinic acid or 2-aminobenzoic acid. Polyhedron 151, 452–457. 10.1016/j.poly.2018.05.057

[B48] Ishikawa N. Sugita M. Okubo T. Tanaka N. Iino T. Kaizu Y. (2003). Determination of ligand-field parameters and f-electronic structures of double-decker bis (phthalocyaninato) lanthanide complexes. Inorg. Chem. 42, 2440–2446. 10.1021/ic026295u 12665381

[B49] Jin J. Wang X. Li Y. Chi Y. Niu S. (2012). Synthesis, structure, and photophysical property of series of Ln (III) coordination polymers with different carboxylato ligands (Ln = Sm, Eu). Struct. Chem. 23, 1523–1531. 10.1007/s11224-012-9957-6

[B50] Kahn O. (1993). Molecular Magnetism. New York, NY: VCH Publishers, Inc.(USA).

[B51] Katkova M. A. Bochkarev M. N. (2010). New trends in design of electroluminescent rare earth metallo-complexes for OLEDs. Dalton Trans. 39, 6599–6612. 10.1039/c001152e 20390195

[B52] Kenyon A. (2002). Recent developments in rare-earth doped materials for optoelectronics. Prog. Quant. Electron. 26, 225–284. 10.1016/S0079-6727(02)00014-9

[B53] Kettle S. F. A. (2013). Physical Inorganic Chemistry: A Coordination Chemistry Approach. Heidelberg: Springer.

[B54] Kido J. Okamoto Y. (2002). Organo lanthanide metal complexes for electroluminescent materials. Chem. Rev. 102, 2357–2368. 10.1021/cr010448y 12059271

[B55] Li Z. Y. Cao Y. Q. Li J.-Y. Zhang X. F. Zhai B. Zhang C. (2017). Three types of lanthanide coordination polymers with methylmalonate and isonicotinate as coligands: structures, luminescence, and magnetic properties. Cryst. Growth Des. 17, 6752–6761. 10.1021/acs.cgd.7b01341

[B56] Marrot F. Trombe J. C. (1994). Synthesis, characterization and structure of a diaqua lanthanum bimalonate malonate monohydrate. Polyhedron 13, 1931–1935. 10.1016/0277-5387(94)80017-0

[B57] Mathew V. Jacob S. Xavier L. Abraham K. (2012). Spectroscopic studies of gel-grown lanthanum malonate crystals. J. Rare Earth. 30, 245–249. 10.1016/S1002-0721(12)60039-8

[B58] Muraishi K. Yokobayashi H. Nagase K. (1991). Systematics on the thermal reactions of lanthanide malonates Ln_2_(C_3_H_2_O_4_)_3_·nH_2_O in the solid state. Thermochim. Acta. 182, 209–217. 10.1016/0040-6031(91)80006-5

[B59] Pagis C. Ferbinteanu M. Rothenberg G. Tanase S. (2016). Lanthanide-based metal organic frameworks: synthetic strategies and catalytic applications. ACS Catal. 6, 6063–6072. 10.1021/acscatal.6b01935

[B60] Plush S. E. Gunnlaugsson T. (2007). Luminescent Sensing of Dicarboxylates in Water by a bismacrocyclic dinuclear Eu(III) Conjugate. Org. Lett. 9, 1919–1922. 10.1021/ol070339r 17447771

[B61] Rahahlia N. Benmerad B. Guehria-Laïdoudi A. Dahaoui S. Lecomte C. (2007). Three-dimensional ionic frameworks built up from La(III) and Ce(III) succinates. J. Mol. Struct. 833, 42–48. 10.1016/j.molstruc.2006.08.029

[B62] Räsänen M. Takalo H. Rosenberg J. Mäkelä J. Haapakka K. Kankare J. (2014). Study on photophysical properties of Eu (III) complexes with aromatic β-diketones–Role of charge transfer states in the energy migration. J. Lumin. 146, 211–217. 10.1016/j.jlumin.2013.09.076

[B63] Reineke T. M. Eddaoudi M. O'keeffe M. Yaghi O. M. (1999). A microporous lanthanide–organic framework. Angew. Chem. Int. Ed. 38, 2590–2594. 10.1002/(SICI)1521-3773(19990903)38:17<2590::AID-ANIE2590>3.0.CO;2-H 10508349

[B64] Reisfeld R. (2015). Optical properties of lanthanides in condensed phase, theory and applications. AIMS Mater. Sci. 2, 37–60. 10.3934/matersci.2015.2.37

[B65] Reisfeld R. Jorgensen C. K. (2012). Lasers and Excited States of Rare Earths. Springer Science & Business Media.

[B66] Rodríguez-Martín Y. Hernández-Molina M. Delgado F. S. Pasán J. Ruiz-Pérez C. Sanchiz J. (2002). Structural versatility of the malonate ligand as a tool for crystal engineering in the design of molecular magnets. Cryst. Eng. Comm. 4, 522–535. 10.1039/B202166H

[B67] Roy S. Chakraborty A. Maji T. K. (2014). Lanthanide–organic frameworks for gas storage and as magneto-luminescent materials. Coord. Chem. Rev. 273, 139–164. 10.1016/j.ccr.2014.03.035

[B68] Seidel C. Lorbeer C. Cybinska J. Mudring A. V. Ruschewitz U. (2012). Lanthanide coordination polymers with tetrafluoroterephthalate as a bridging ligand: thermal and optical properties. Inorg. Chem. 51, 4679–4688. 10.1021/ic202655d 22443467

[B69] Sharif S. Khan I. U. Sahin O. Ahmad S. Büyükgüngör O. Ali S. (2012). Synthesis and crystal structures of a lanthanum(III) 1D polymer and a mixed-ligand cerium (III) binuclear complex derived from pyridine-2, 6-dicaboxylic acid. J. Inorg. Organomet. Polym. 22, 1165–1173. 10.1007/s10904-012-9715-7

[B70] Sharma G. Narula A. K. (2015). Synthesis and optoelectronic properties of three Eu(III)-dipicolinate complexes based on α-picolinic acid, 2-aminopyridine and 2-hydroxypyridine as secondary ligands. J. Mater. Sci. Mater. Electron. 26, 1009–1017. 10.1007/s10854-014-2497-7

[B71] Sheldrick G. M. (2008). A short history of SHELX. Acta Cryst. A. Found. Crystallogr. 64, 112–122. 10.1107/S0108767307043930 18156677

[B72] Sheldrick G. M. (2014). “SADABS v. 2014/5”. Bruker/Siemens Area Detector Absorption Correction Program. Madison, WI: Bruker AXS Inc.

[B73] Sheldrick G. M. (2015). Crystal structure refinement with SHELXL. Acta Crystallogr. Sect. C. Struct. Chem. 71, 3–8. 10.1107/S2053229614024218 25567568 PMC4294323

[B74] Shibasaki M. Yoshikawa N. (2002). Lanthanide complexes in multifunctional asymmetric catalysis. Chem. Rev. 102, 2187–2210. 10.1021/cr010297z 12059266

[B75] Silva P. Fernandes J. A. Almeida Paz F. A. (2010). Catena-poly [[triaquachlorido-μ_3_-malonato-cerium(III)] hemihydrate]. Acta Crystallogr. Sect. E Struct. Rep. Online. 66, m1514–m1515. 10.1107/S1600536810044727 PMC3011794 21589218

[B76] Sinha S. P. (2012). Systematics and the Properties of the Lanthanides. Dordrecht: Springer Science & Business Media

[B77] Sun Q. Yan P. Niu W. Chu W. Yao X. An G. (2015). NIR luminescence of a series of benzoyltrifluoroacetone erbium complexes. RSC Adv. 5, 65856–65861. 10.1039/C5RA12954K

[B78] Terai T. Kikuchi K. Iwasawa S.-Y. Kawabe T. Hirata Y. Urano Y.. (2006). Modulation of luminescence intensity of lanthanide complexes by photoinduced electron transfer and its application to a long-lived protease probe. J. Am. Chem. Soc. 128, 6938–6946. 10.1021/ja060729t 16719474

[B79] Thirumurugan A. Natarajan S. (2004). Inorganic– organic hybrid compounds: synthesis and structures of new metal organic polymers synthesized in the presence of mixed dicarboxylates. Eur. J. Inorg. Chem. 2004, 762–770. 10.1002/ejic.200300594

[B80] Tian J. Li B. Zhang X. Li X. Li X. Zhang J. (2013). Three novel 1D lanthanide-carboxylate polymeric complexes: syntheses, crystal structures and magnetic analyses. Dalton Trans. 42, 8504–8511. 10.1039/c3dt50782c 23629687

[B81] Van Vleck J. (1932). The Theory of Electronic and Magnetic Susceptibility. London: Oxford University Press.

[B82] Wahsner J. Gale E. M. Rodríguez-Rodríguez A. Caravan P. (2019). Chemistry of MRI contrast agents: current challenges and new frontiers. Chem. Rev. 119, 957–1057. 10.1021/acs.chemrev.8b00363 30350585 PMC6516866

[B83] Wang C. G. Xing Y. H. Li Z. P. Li J. Zeng X. Q. Ge M. F. (2009). Synthesis, crystal structures and properties of a series of three-dimensional lanthanide coordination polymers with the rigid and flexible mixed dicarboxylate ligands of 1, 4-benzene dicarboxylic acid and succinic acid. J. Mol. Struct. 921, 126–131. 10.1016/j.molstruc.2008.12.057

[B84] Wenmei X. Qiguang W. Lan Y. Rudong Y. (1992). Synthesis, characterization and crystal structure of tri-aquo bimalonate malonate samarium (III) monohydrate. Polyhedron 11, 2051–2054. 10.1016/S0277-5387(00)83161-7

[B85] Woodruff D. N. Winpenny R. E. Layfield R. A. (2013). Lanthanide single-molecule magnets. Chem. Rev. 113, 5110–5148. 10.1021/cr400018q 23550940

[B86] Yan B. Bai Y. Chen Z. (2005). Synthesis, structure and luminescence of novel 1D chain coordination polymers [Ln(isophth)(Hisophth)(H_2_O)_4_·4H_2_O]_n_ (Ln = Sm, Dy). J. Mol. Struct. 741, 141–147. 10.1016/j.molstruc.2005.02.004

[B87] Zhang C. Z. Mao H. Y. Wang Y. L. Zhang H. Y. Tao J. C. (2007). Syntheses of two new hybrid metal-organic polymers using flexible aliphatic dicarboxylates and pyrazine: crystal structures and magnetic studies. J. Phys. Chem. Solids. 68, 236–242. 10.1016/j.jpcs.2006.11.001

[B88] Zhao B. Chen X. Y. Cheng P. Liao D. Z. Yan S.-P. Jiang Z. H. (2004). Coordination polymers containing 1D channels as selective luminescent probes. J. Am. Chem. Soc. 126, 15394–15395. 10.1021/ja047141b 15563162

[B89] Zhu W. H. Li S. Gao C. Xiong X. Zhang Y. Liu L.. (2016). Lanthanide dinuclear complexes constructed from mixed oxygen-donor ligands: the effect of substituent positions of the neutral ligand on the magnetic dynamics in Dy analogues. Dalton Trans. 45, 4614–4621. 10.1039/C5DT04850H 26847996

[B90] Zhuravlev K. P. Tsaryuk V. I. Pekareva I. S. Sokolnicki J. Klemenkova Z. S. (2011). Europium and terbium ortho-, meta-, and para-methoxybenzoates: Structural peculiarities, luminescence, and energy transfer. J. Photoch. Photobio. A 219, 139–147. 10.1016/j.jphotochem.2011.02.003

