# Supplementary material for: Synthesis, Thermal, Structural Analyses, and Photoluminescent Properties of a New Family of Malonate-Containing Lanthanide(III) Coordination Polymers
Source: Front Chem. 2019 Apr 30;7:260. doi: 10.3389/fchem.2019.00260 (PMC6503220; doi:10.3389/fchem.2019.00260)
Supplement: Supplementary file 1 [file Data_Sheet_1.pdf]

Supporting Information

Synthesis, Thermal, Structural Analyses and Photoluminescent Properties of a New Family of Malonate-containing Lanthanide(III) Coordination Polymers

Sajjad Hussain,[*a, b] Xuenian Chen,[*a] William T. A. Harrison,[c] Saeed Ahmad,[d] Mark R.J. Elsegood,[e] Islam Ullah khan,[f] Shabbir Muhammad,[g]

[a]*School of Chemistry and Chemical Engineering, Henan Key Laboratory of Boron Chemistry and Advanced Energy Materials, Henan Normal University, Xinxiang 453007, China*

E-mail: xnchen@htu.edu.cn(X.C), sajjaduet07@yahoo.com(S.H)

[b]*Department of Chemistry, Mohi Ud Din Islamic University Nerian Sharif, Azad Jammu and Kashmir, Pakistan*

[c]*Department of Chemistry, University of Aberdeen, Aberdeen AB24 3UE, Scotland*

[d]*Department of Chemistry, College of Sciences and Humanities, Prince Sattam Bin Abdul Aziz University, Al-Kharj 11942, Saudi Arabia*

[e]*Chemistry Department, Loughborough University, Loughborough LE11 3TU, United Kingdom*

[f]*Department of Chemistry, Government College University, Lahore, Pakistan*

[g]*Department of Physics, College of Science, King Khalid University, Abha, Saudi Arabia*

Table S1. Selected bond lengths (Å), bond angles (°) and torsion angles (°) for **2**

Tb1–O1	2.315(2)	O1–Tb1– O1^{i}	115.95(17)
Tb1–O2^{i}	2.364(2)	O1–Tb1–O2	77.09(9)
Tb1–O3	2.442(3)	O1–Tb1–O5	76.23(8) 4
C1–O1	1.249(3)	O2–Tb1–O3	80.21(9)
Tb1–O4	2.404(2)	O2–Tb1– O4	112.68(9)
Tb1–O5	2.401(3)	O4–Tb1–O3	144.42(7)
C1–O2	1.262(4)	O5–Tb1–O3	117.84(12)
O1–C1–C2–C1^{ii}	125.7(3)	O2–C1–C2–C1^{ii}	–54.7(5)

Symmetry codes: (i) *x*–½, 1–*y*, *z*–½; (ii) –*x*+2, *y*, *z*; (iii) *x*, *y*–1, *z*; (iv) -*x*+3/2, -*y*+2, *z*+1/2.

Table S2. Selected bond lengths (Å), bond angles (°) and torsion angles (°) for **3**

Ho1–O1	2.297(3)	O1–Ho1–O1^{i}	115.9(2)
Ho1–O2^{i}	2.343(3)	O1–Ho1–O2	76.77(11)
Ho1–O3	2.410(4)	O1–Ho1–O5	76.25(10)
Ho1–O4	2.374(3)	O2–Ho1–O3	80.02(12)
Ho1–O5	2.378(5)	O2–Ho1–O4	112.90(11)
C1–O1	1.248(5)	O4 –Ho1–O3	144.49(9)
C1–O2	1.260(5)	O5–Ho1–O3	117.68(16)
O1–C1–C2–C1^{ii}	–126.1(4)	O2–C1–C2–C1^{ii}	54.0(7)

Symmetry codes: (i) ½+*x*, 1–*y*, ½+*z*; (ii) –*x*, *y*, *z;*(iii) *x*, *y*+1, *z*; (iv) ½–*x*, –*y*, *z*–½;.

Table S3. Selected bond lengths (Å), bond angles (°) and torsion angles (°) for **4**

Er1–O1	2.414(3)	O1–Er1–O1^{i}	128.23(18)
Er1–O3	2.254(4)	O3–Er1–O1	71.23(12)
Er1–O4^{i}	2.355(4)	O3–Er1–O4	75.10(13)
Er1–O5	2.314(4)	O3–Er1–O5	147.17(14)
C1–O1	1.242(5)	O4–Er1–O1	130.96(14)
C1–O2	1.286(6)	O5–Er1–O4	69.21(13
C3–O4	1.247(6)		
C3–O3	1.252(6)		
O1–C1–C2–C3	29.0(8)	O2–C1–C2–C3	–152.4(5)
C1–C2–C3–O4	154.3(5)	C1–C2–C3–O3	–27.8(7)

Symmetry codes: (i) ¼–*x*, *y*–¼, *z*–¼; (ii) 1–*x*, 1–*y*, *z*; (iii) *x*–¼, 1¼–*y*, *z*–¼; (iv) ¼+*x*, ¾–*y*, *z*–¼; (v) ¾–*x*, ¼+*y*, *z*–¼.

Table S4. Selected bond lengths (Å) and bond angles (°) for **5**

Eu1–O1	2.388(2)	O1–Eu1–O3	71.94(7)
Eu1–O3	2.405(2)	O1–Eu1–O5	147.83(7)
Eu1–O4[i]	2.339(2)	O1–Eu1–O9	122.75(7)
Eu1–O5	2.438(2)	O1–Eu1–O10	78.70(7)
Eu1–O9	2.579(2)	O3–Eu1–O5	82.73(7)
Eu1–O10	2.591(2)	O4–Eu1–O5	88.51(8)
Eu1–O17	2.417(2)	O4–Eu1–O9	140.16(8)
Eu1–O18	2.451(2)	O5–Eu1–O10	112.73(7)
Eu1–O19	2.475(2)	O18–Eu1–O10	84.61(8)
Eu2–O5	2.694(2)	O13–Eu2–O15	93.78(8)
Eu2–O6	2.493(2)	O13–Eu2–O14	138.35(7)
Eu2–O9	2.430(2)	O13–Eu2–O22	78.52(8)
Eu2–O13	2.342(2)	O14–Eu2–O20	69.05(7)
Eu2–O14[ii]	2.4055(19)	O14–Eu2–O22	135.45(7)
Eu2–O15[ii]	2.387(2)	O15–Eu2–O14	72.32(7)
Eu2–O20	2.442(2)	O20–Eu2–O21	128.78(8
Eu2–O21	2.508(2)	O13–Eu2–Eu1	119.94(6)
Eu2–O22	2.411(2)		
C1–O2	1.247(3)	C1–O1	1.268(3)
C3–O4	1.249(3)	C3–O3	1.263(4)
C4–O5	1.259(3)	C4–O6	1.265(3)
C6–O7	1.209(4)	C6–O8	1.318(4)
C7–O10	1.254(3)	C7–O9	1.267(3)
C9–O12	1.212(4)	C9–O11	1.316(4)
C10–O13	1.253(3)	C10–O14	1.266(3)
C12–O15	1.260(3)	C12–O16	1.260(3)

Symmetry codes: (i) ½–*x*, *y*–½, 1½–*z*; (ii) –*x*–½, ½+*y*, 1½–*z*.

Table S5. Hydrogen bonds for **1**

D—H···*A*	*D*—*H*	*H*···*A*	*D*···*A*	*D*—*H*···*A*
O3–H3···O4^{iii}	0.82(3)	2.33(4)	3.103(6)	157(6)
O4–H4A···O6	0.82(3)	2.01(3)	2.803(3)	163(6)
O4–H4B···O7^{iv}	0.82(3)	1.92(3)	2.714(6)	165(6)
O5–H5···O2	0.83(3)	2.06(5)	2.778(3)	144(7)

Symmetry codes: (i) *x*–½, 1–*y*, *z*–½; (ii) -*x*+2, *y*, *z*; (iii) *x*, *y*-1, *z*; (iv) -*x*+3/2, -*y*+2, *z*+1/2.

Table S6. Hydrogen bonds for **2**

D—H···*A*	*D*—*H*	*H*···*A*	*D*···*A*	*D*—*H*···*A*
O3–H3···O4^{iii}	0.81(2)	2.38(3)	3.104(5)	149(4)
O4–H4A···O6	0.81(2)	1.99(2)	2.795(3)	170(5)
O4–H4B···O7^{iv}	0.81(2)	1.91(2)	2.722(4)	173(5)
O5–H5···O2	0.81(2)	2.03(3)	2.775(3)	153(5)

Symmetry codes: (i) *x*–½, 1–*y*, *z*–½; (ii) –*x*+2, *y*, *z*; (iii) *x*, *y*–1, *z*; (iv) -*x*+3/2, -*y*+2, *z*+1/2.

Table S7. Hydrogen bonds for **3**

D—H···*A*	*D*—*H*	*H*···*A*	*D*···*A*	*D*—*H*···*A*
O3–H3···O4^{iii}	0.81(3)	2.36(3)	3.111(6)	155(5)
O4–H4A···O6	0.82(3)	2.01(3)	2.797(3)	160(7)
O4–H4B···O7^{iv}	0.83(3)	1.90(3)	2.724(5)	175(6)
O5–H5···O2	0.83(3)	1.99(4)	2.767(3)	156(7)

Symmetry codes: (i) ½+*x*, 1–*y*, ½+*z*; (ii) –*x*, *y*, *z;*(iii) *x*, *y*+1, *z*; (iv) ½–*x*, –*y*, *z*–½;.

Table S8. Hydrogen bonds for **4**

D—H···*A*	*D*—*H*	*H*···*A*	*D*···*A*	*D*—*H*···*A*
O2–H2···O2^{ii}	0.83	1.64	2.453(7)	167
O5–H5A···O2^{iii}	0.89	2.05	2.903(5)	160
O5–H5B···O3^{iv}	0.85	2.00	2.731(6)	143
O5–H5B···O1^{v}	0.85	2.27	2.941(5)	135

Symmetry codes: (i) ¼–*x*, *y*–¼, *z*–¼; (ii) 1–*x*, 1–*y*, *z*; (iii) *x*–¼, 1¼–*y*, *z*–¼; (iv) ¼+*x*, ¾–*y*, *z*–¼; (v) ¾–*x*, ¼+*y*, *z*–¼.

Table S9. Hydrogen bonds for **9**

D—H···*A*	*D*—*H*	*H*···*A*	*D*···*A*	*D*—*H*···*A*
O8–H8···O24[iii]	0.86	1.77	2.638(4)	176
O11–H11···O6[iv]	0.83	1.76	2.569(3)	163
O17–H17A···O25[iv]	0.83(2)	1.92(2)	2.736(4)	165(4)
O17–H17B···O16[iii]	0.84(2)	1.91(2)	2.740(3)	172(4)
O18–H18A···O15[iii]	0.84(2)	1.98(2)	2.782(3)	160(4)
O18–H18B···O14[ii]	0.85(2)	1.87(2)	2.703(3)	167(4)
O19–H19A···O12[i]	0.83(2)	2.19(2)	3.017(4)	173(4)
O19–H19B···O16[i]	0.83(2)	1.95(2)	2.772(3)	171(4)
O20–H20A···O7[v]	0.84(2)	2.00(1)	2.834(3)	172(4)
O20–H20B···O2[vi]	0.84(1)	1.95(2)	2.758(3)	161(4)
O21–H21A···O2[vii]	0.84(1)	1.90(1)	2.736(3)	179(4)
O21–H21B···O26	0.84(1)	2.10(2)	2.861(6)	151(4)
O21–H21B···O26X	0.84(1)	1.99(1)	2.826(9)	178(4)
O22–H22A···O1[vii]	0.84(1)	1.91(1)	2.740(3)	169(4)
O22–H22B···O3	0.84(1)	1.95(1)	2.769(3)	168(4)
O23–H23A···O10[viii]	0.84(2)	2.03(2)	2.851(3)	167(5)
O23–H23B···O21	0.83(2)	2.04(2)	2.846(4)	166(5)
O24–H24A···O25	0.84(2)	2.06(3)	2.785(4)	145(4)
O24–H24B···O16[ix]	0.84(2)	2.19(3)	2.979(4)	158(5)
O25–H25A···O23[x]	0.83(2)	2.03(3)	2.813(4)	159(5)
O25–H25B···O23	0.85(2)	1.93(3)	2.746(4)	161(5)

Symmetry codes: (i) ½–*x*, *y*–½, 1½–*z*; (ii) –*x*–½, ½+*y*, 1½–*z*; (iii) *x*, 1+*y*, *z*; (iv) ½+*x*, 1½–*y*, ½+*z*; (v) –½–*x*, *y*–½, 1½–*z*; (vi) *x*–1, *y*, *z*; (vii) ½–*x*, *y*–½, 1½–*z*; (viii) *x*–½, 1½–*y*, *z*–½; (ix) *x*–½, ½–*y*, *z*–½ ; (x) –*x*, 1–*y*, 1–*z*.

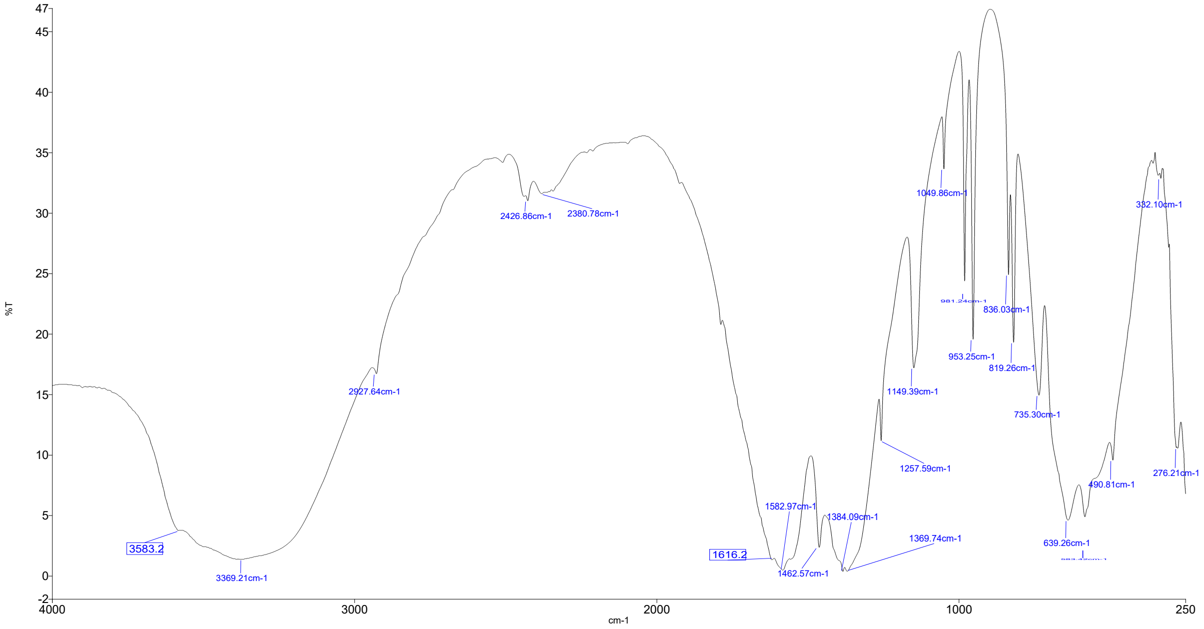

Fig. S1. IR spectrum of $\{[Gd(C_3H_2O_4)(H_2O)_4]\cdot NO_3\}_n$ (**1**)

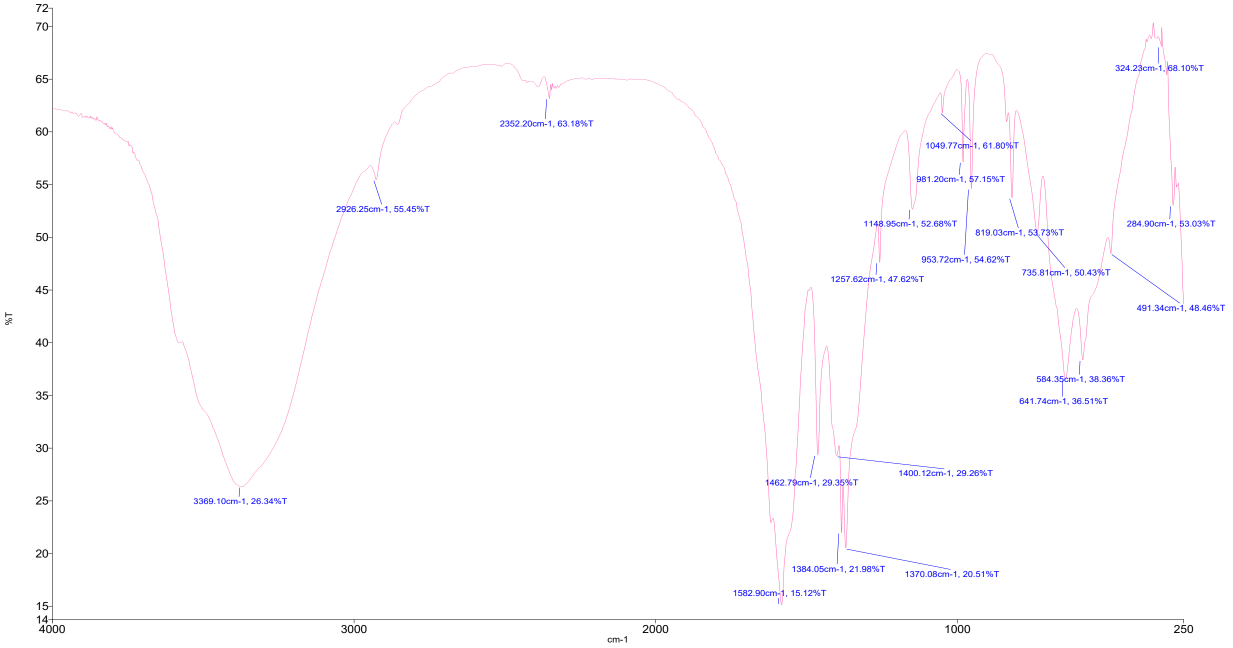

Fig. S2. IR spectrum of $\{[Tb(C_3H_2O_4)(H_2O)_4]\cdot NO_3\}_n$ (**2**)

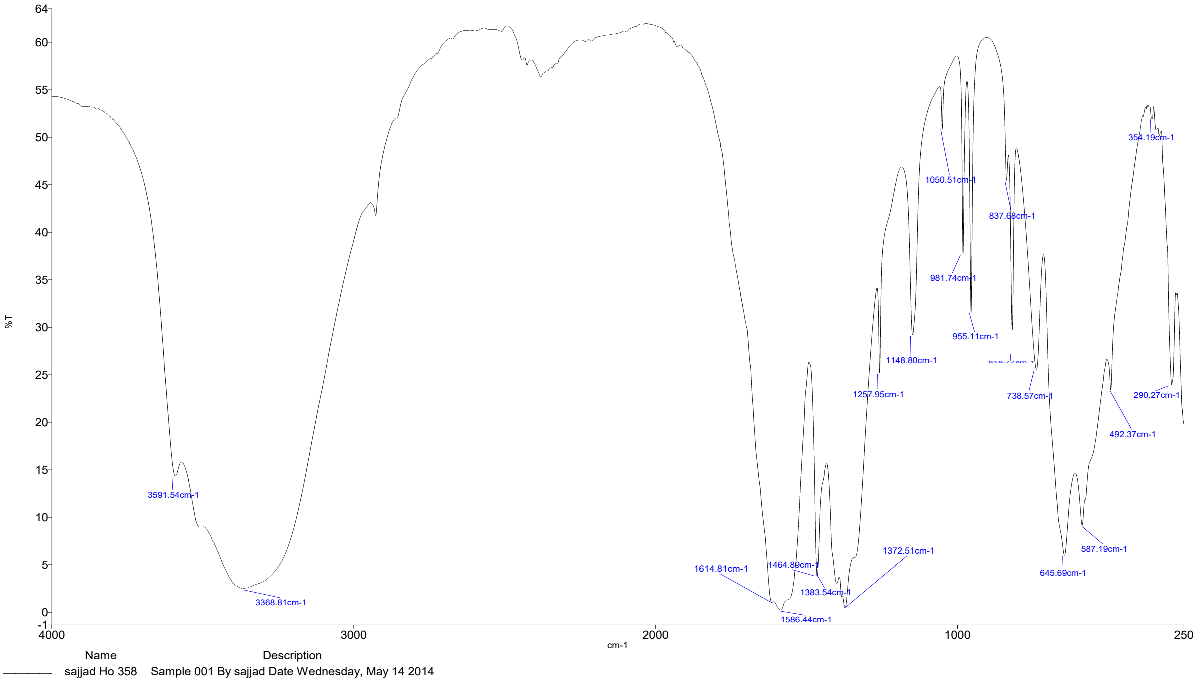

Fig. S3. IR spectrum of $\{[Ho(C_3H_2O_4)(H_2O)_4]\cdot NO_3\}_n$ (**3**)

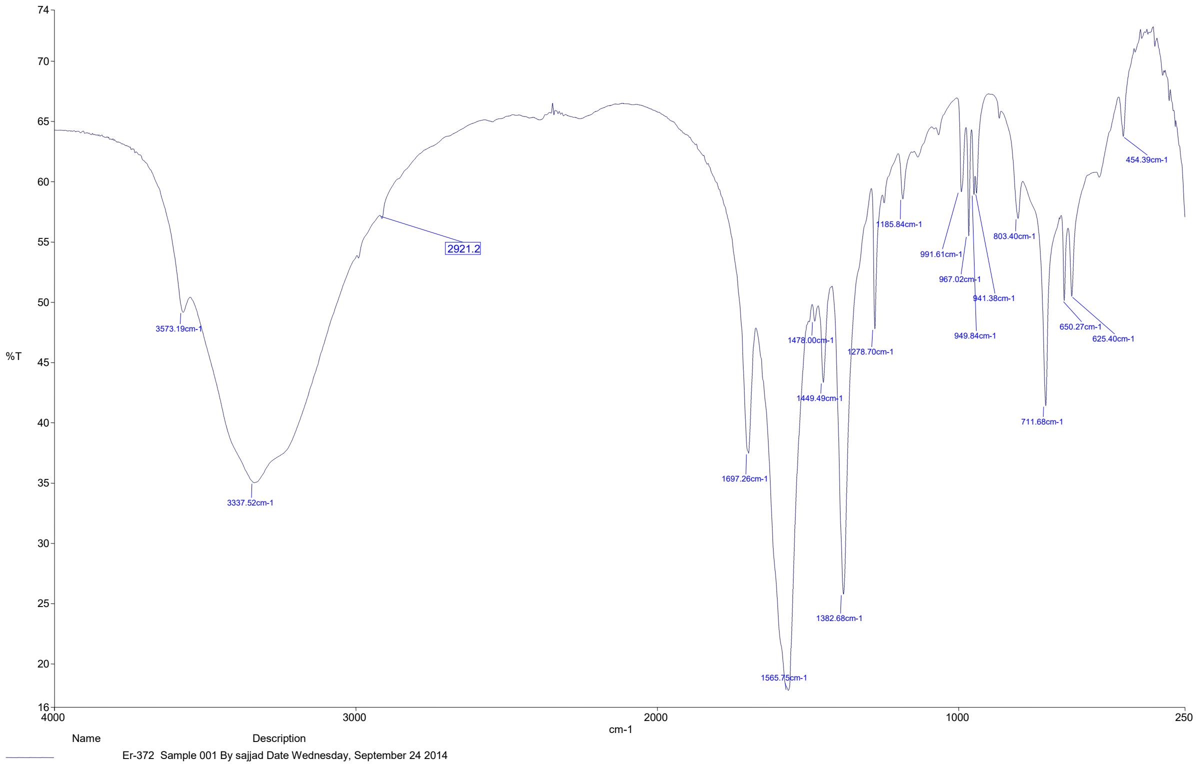

Fig. S4. IR spectrum of $[Er(C_3H_2O_4)(C_3H_3O_4)(H_2O)_2]_n$ (**4**)

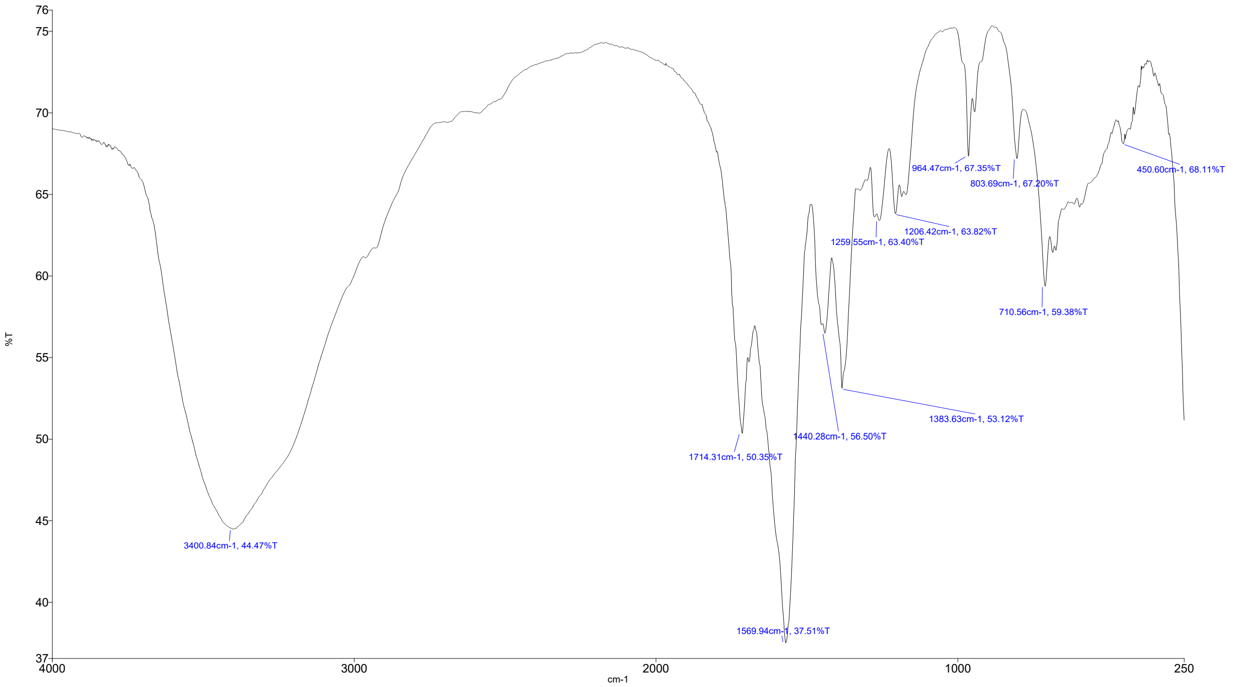

Fig. S5. IR spectrum of $\{[Eu_2(C_3H_2O_4)_2(C_3H_3O_4)_2(H_2O)_6]\cdot 4H_2O\}_n$ (**5**)

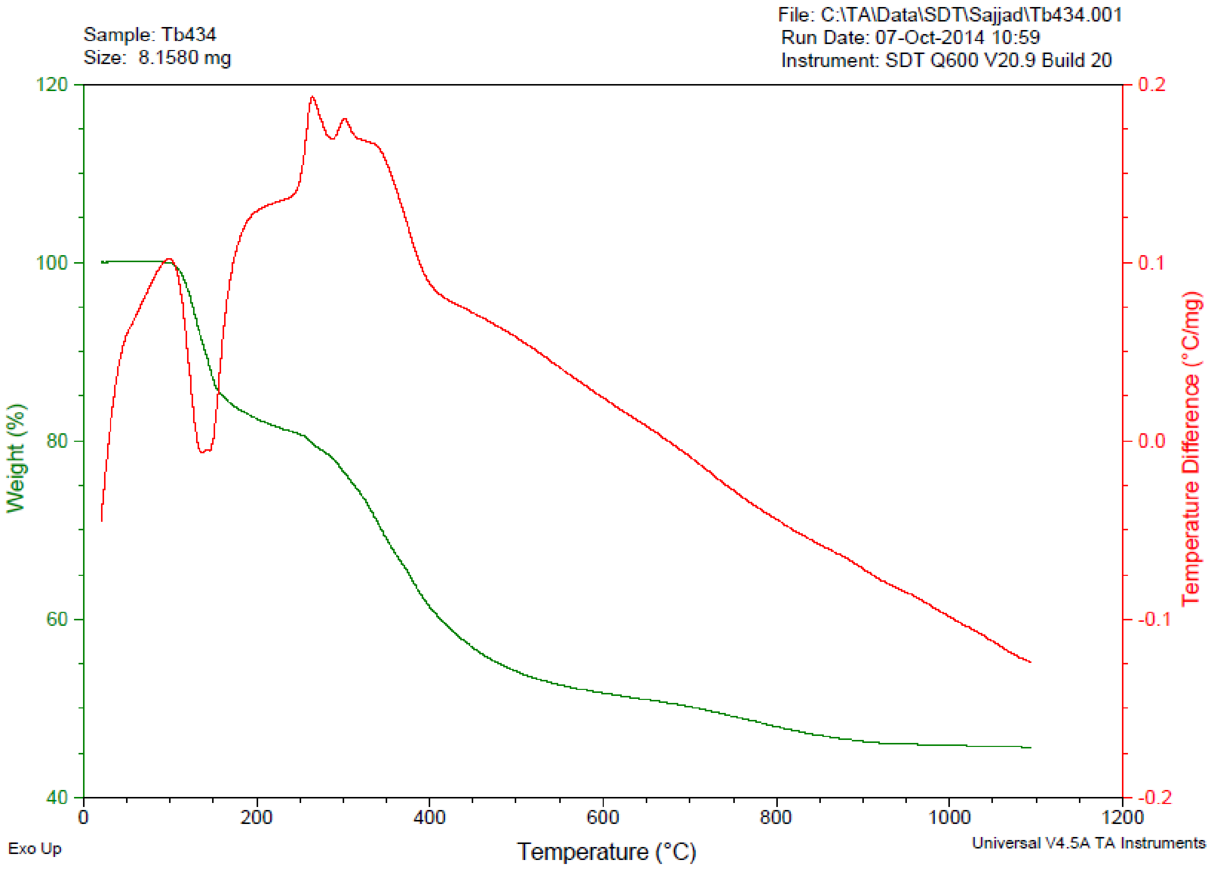

Fig. S6. TGA and DSC curves of {[Tb($C_3H_2O_4$)(H_2O)$_4$]·NO_3}$_n$ (**2**)

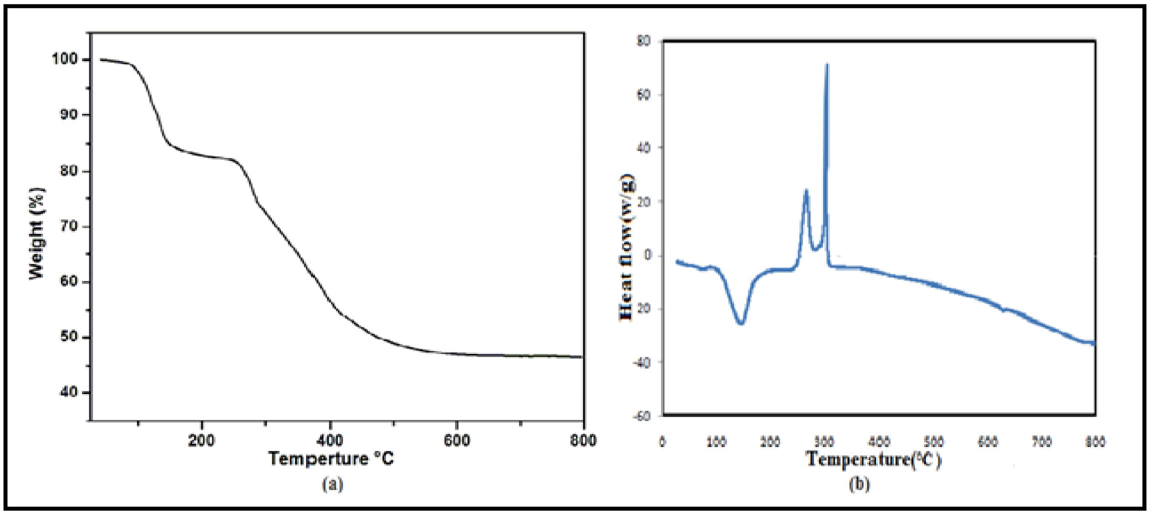

Fig. S7. TGA and DSC curves of {[Ho($C_3H_2O_4$)(H_2O)$_4$]·NO_3}$_n$ (**3**)

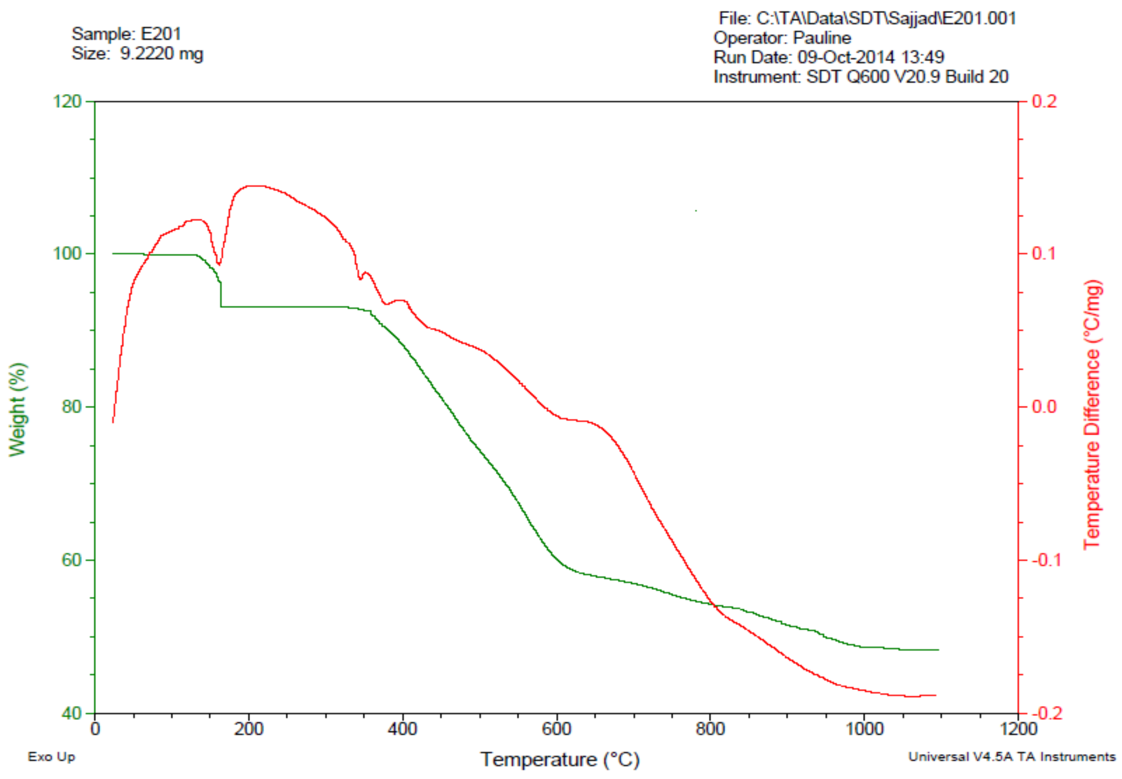

Fig. S8. TGA and DSC curves of $[Er(C_3H_2O_4)(C_3H_3O_4)(H_2O)_2]_n$ **(4)**

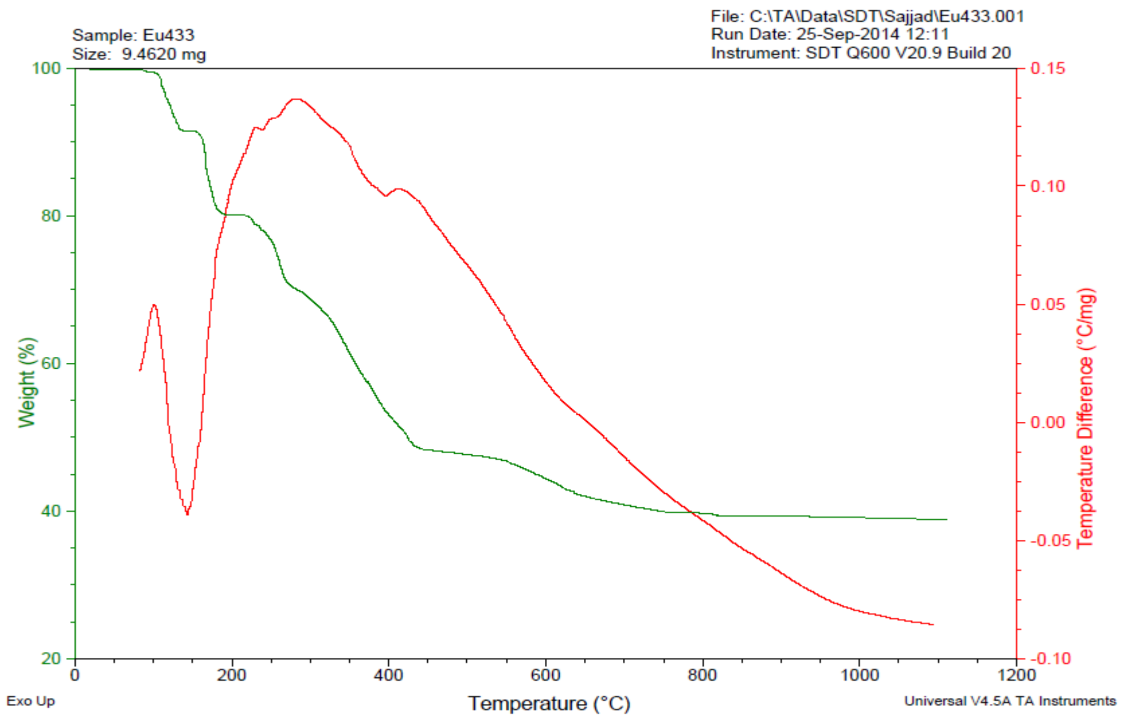

Fig. S9. TGA and DSC curves of $\{[Eu_2(C_3H_2O_4)_2(C_3H_3O_4)_2(H_2O)_6]\cdot 4H_2O\}_n$(**5**)

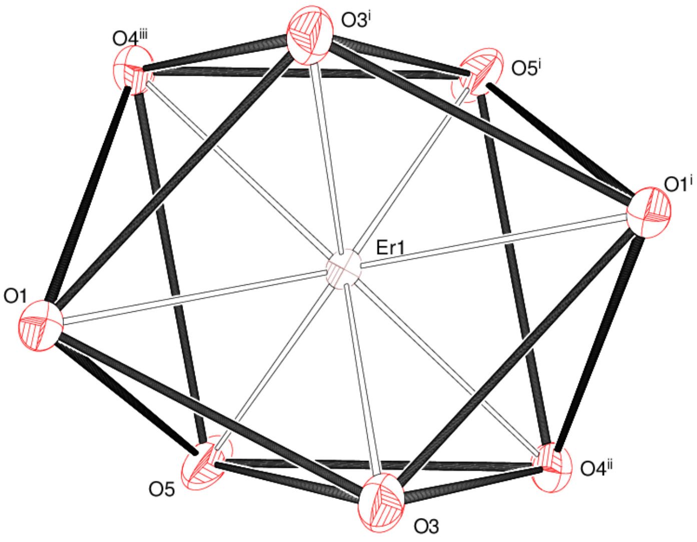

Fig. S10. The square antiprismatic metal coordination geometry in **4**

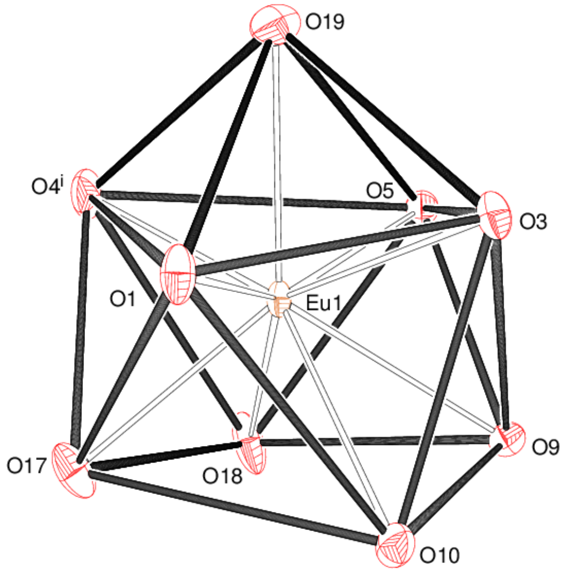

Fig. S11. The capped square antiprismatic metal coordination geometry for Eu1 in **5** (Eu2 has a similar environment).

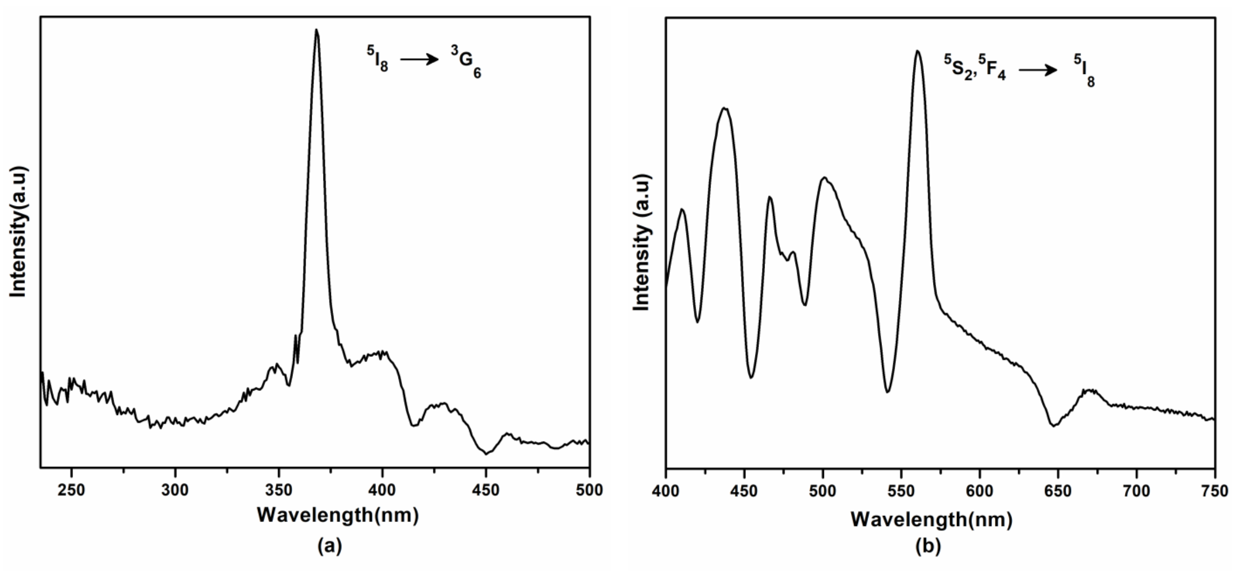

Fig. S 12 (a) Solid state excitation spectrum of **3** to monitor emission wave length at 570 nm. (b) Solid state emission spectrum of **3** excited at 370 nm.

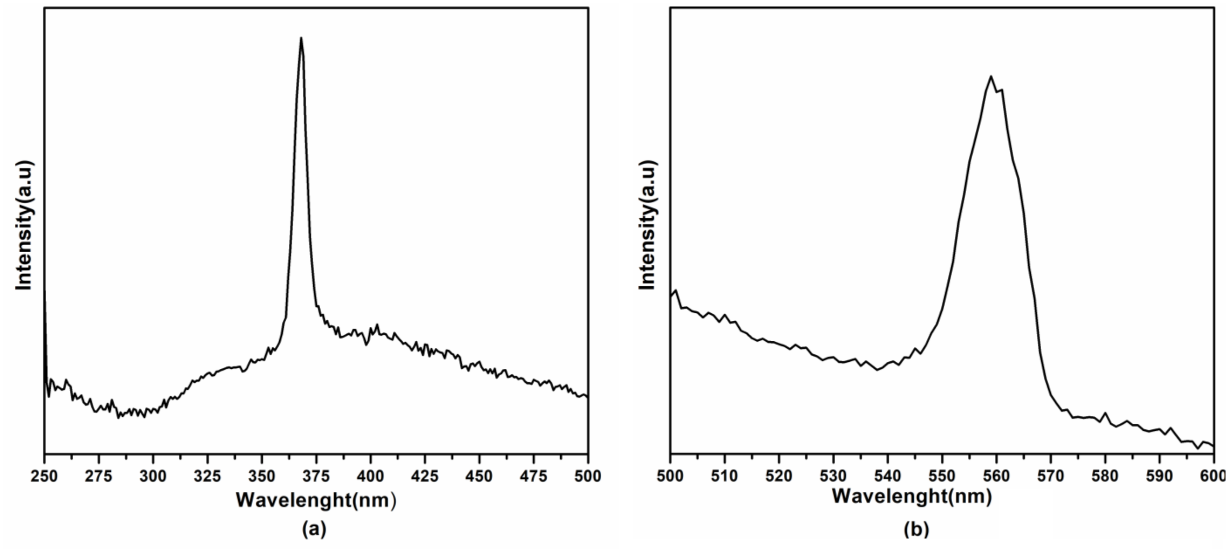

Fig. S 13 (a) Solid state excitation spectrum of **3** to monitor emission wave length at 570 nm. (b) Solid state emission spectrum of **3** excited at 370 nm.

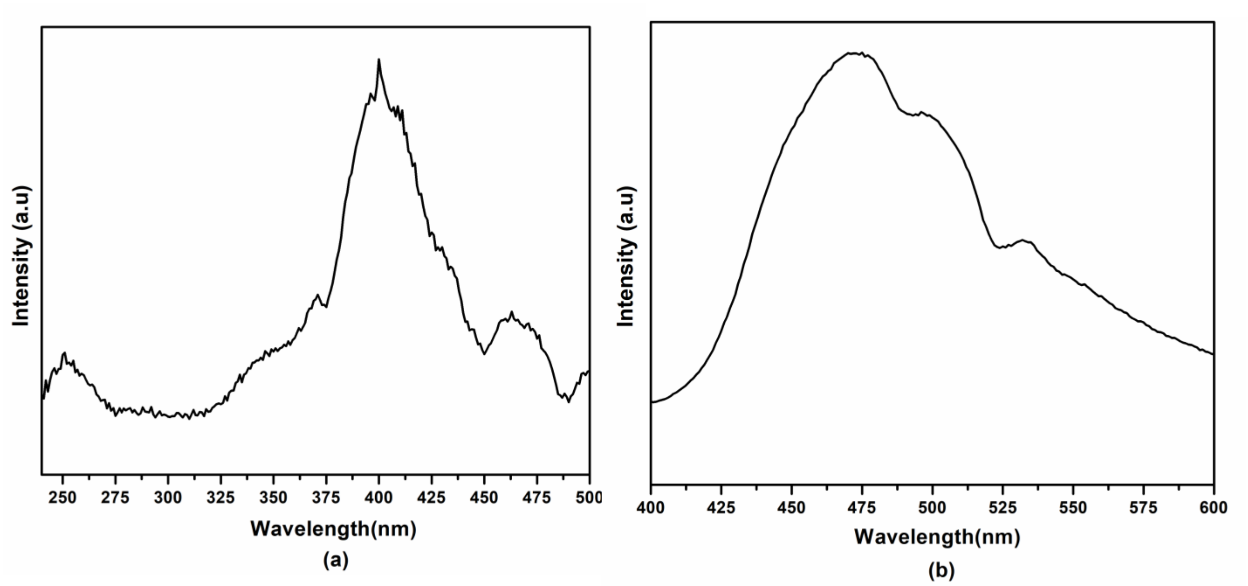

Fig. S 14 **(**a) Solid state excitation spectrum of **4** to monitor emission wave length at 570 nm. (b) Solid state emission spectrum of **4** excited at 380 nm.

Magnetic susceptibility

The magnetic susceptibility were measured at room temperature by Evans balance Mark 1 Sherwood Scientific Ltd, Cambridge UK, CB1 8DH. The employed calibrant was $Hg[Co(SCN)_4]$, for which the susceptibility was assumed to be 1.644×10^{-5} cm^3g^{-1}.[1] The diamagnetic correction for the constituent atoms was calculated by the using of Pascal constants.[2, 3]

Molar magnetic susceptibility is determined by using the formula

$\chi_M = [CL(R-R_o)M/10^3m]x10^6$ in cgs units

χ_M = molar magnetic susceptibility

C = calibration constant

L = length of the tube filled with sample in cm

R_o= balance reading with empty tube

R = balance reading with filled tube

M = formula weight of sample

m = mass of sample in gram

$$\chi'_M = \chi_M - DC$$

DC = diamagnetic correction

χ'_M = corrected molar susceptibility

The effective magnetic moment values were calculated from by using the relation.

$\mu_{eff} = 2.83\sqrt{(\chi M'T)}.$

Where μ eff is the effective magnetic moment in Bohr magneton and T is the temperature in Kelvin.

Computational Details

The crystal structure of compound **1** was optimized within the periodic boundary conditions using CASTEP module of Materials Studio[4] as shown in Fig. S 15. The total density of states (TDOS), partial density of states (PDOS) and optical properties (dielectric function, conductivity, refractive index, extinction coefficient and solid-state absorption spectrum) were studied by adopting the exchange and correlation potential (DFT/GGA/PBE),[5] which is commonly used function to compute different properties of interests in material science. [5, 6] It introduces a dependence of exchange and correlation energy on the local gradient of the electron density. We applied the ultra-soft pseudo-potentials,[7-9] where the shallow core electrons generally treated like valence states by adding various sets of occupied states in every angular momentum channel as well as improve the precision and transferability of the potentials.[9-12] The sampling of Brillouin Zone (BZ) through the reciprocal-space integration was approximated by using the Monkhorst–Pack scheme. An optimized cutoff energy of 340 eV for plane-wave expansion was used which spontaneously creates the fast Fourier transform (FFT) grid for compound **1**. The ultra-soft pseudo-potential and electronic minimization method representation have been chosen using reciprocal space and density mixing respectively. The updated scheme of Broyden–Fletcher–Goldfarb–Shanno hessian (BFGS)[13] has been employed as minimization technique. The criteria used for the geometry optimization convergence tolerance energy 1×10^{-5} eV/atom, the tolerance of self-consistent field (SCF) convergence was 1×10^{-6} eV/atom, root-mean-square (RMS) force on atom tolerance was set as 0.03 eV/Å, RMS displacement of atoms was 0.001Å and RMS stress tensor was 0.05 GPa for all calculations.

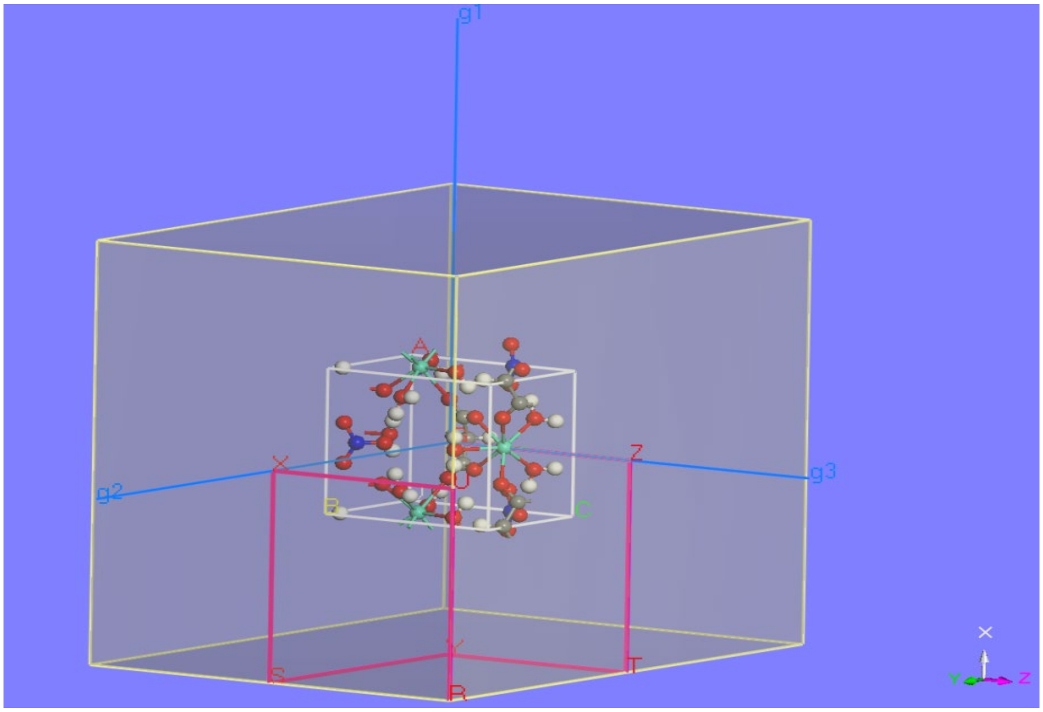

Fig. S15. The optimized geometry of compound **1** within periodic boundary conditions at PBE/GGA level of theory where red color lines represent the high symmetry points of Brillouin Zone.

Table S10. The selected bond lengths (Å) comparison between computationally optimized geometry and experimental single crystal geometry of compound **1**

Bond Lengths (Å)	Experimental (Å)	Calculated (Å)
Gd1–O1	2.330	2.373
Gd1–O2	2.377	2.386
Gd1–O3	2.455	2.620
Gd1–O4	2.415	2.373
Gd1–O5	2.421	2.448
O2– Gd1– O2	73.45	74.69
O2– C2– C1	119.6	120.26
Gd1– O2–C1	132.45	131.35
O2–C1– O1	124.68	123.69

The Computational Geometrical Parameters of Compound 1

A comparison of computationally optimized geometry at PBE/GGA level of theory and the structure obtained by single crystal X-ray is presented in Table S10 and Fig.S13. The central Gd shows different bonds lengths with surrounding O atoms. For instance, the experimental and computed Gd1–O1 bonds are found to be 2.330 Å and 2.373 Å, respectively. Similarly, the bond lengths of Gd1–O2, Gd1–O4, and Gd1–O5 are also found in agreement with computationally calculated bonds. While on the other hands, for Gd1–O3 bond, there is slightly more deviation between experimental and calculated bond lengths which is perhaps due to the electronic repulsion effects for weekly coordinated water molecule. Additionally, the bond angles also shown reasonable agreement among the computationally optimized geometries and experimental single crystal geometries. A comprehensive view of both the geometries (experimental and calculated) as placed side by side is shown in Fig.S13, which indicates that there is no catastrophic deviation between experimental and computational geometries. Thus, the computational methodology might be considered a reliable approach for further exploration of optical parameters.

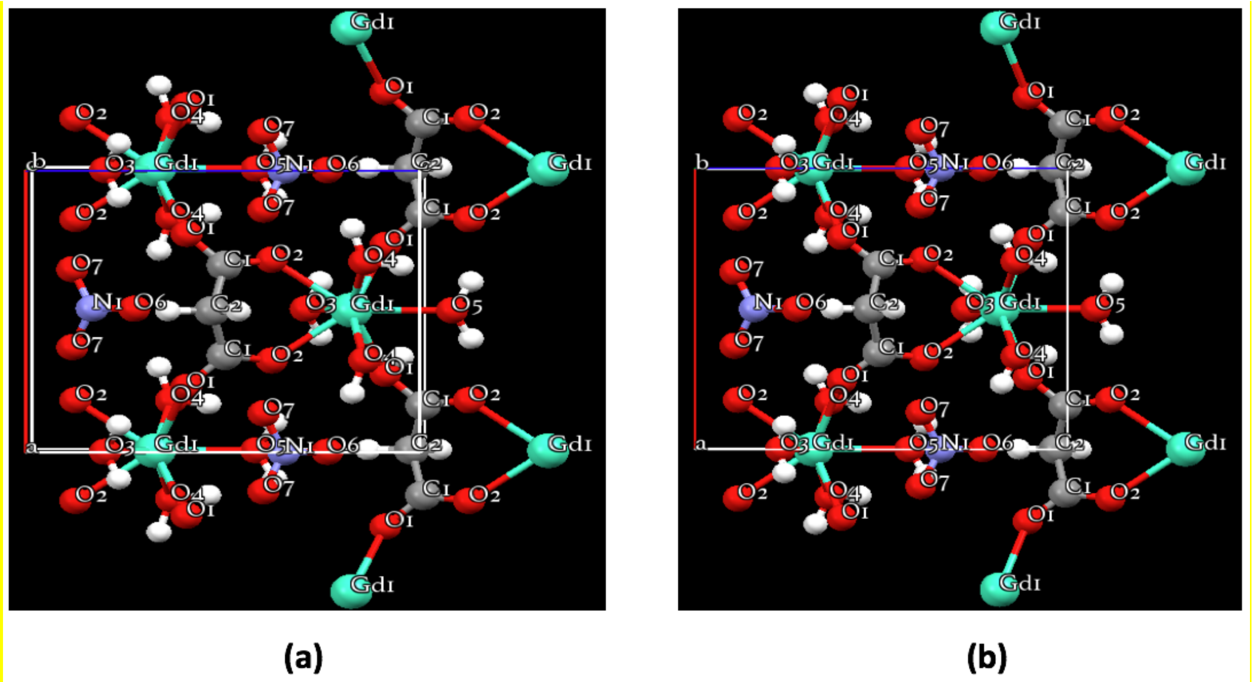

Figure S 16. (a) The computationally optimized geometry within periodic boundary conditions (b) experimental single crystal geometry of compound **1**

References

1. B. N. Figgis, R. Nyholm, D. Nasipuri, B. E. Betts, W. Davey, M. A. P. Hogg, J. E. Spice, E. Boyland, P. Sims, M. M. Coombs, and J. Lamy, *J. Chem. Soc.* 1958, **0,** 4190-4216.
2. O. Kahn, ed., *Molecular Magnetism,* VCH Publishers, New York, 1993.
3. A. Earnshaw, ed., *Introduction to Magnetochemistry*, Academic press, New York 1668.
4. S. J. Clark, M. D. Segall, C. J. Pickard, P. J. Hasnip, M. I. Probert, K. Refson and M. C. Payne, *Z. Kristallogrv.Cryst. Mater* , 2005, **220**, 567-570.
5. J. P. Perdew and Y. Wang, *Phys.Rev.B*, 1992, **45**, 13244.
6. J. P. Perdew, J. A. Chevary, S. H. Vosko, K. A. Jackson, M. R. Pederson, D. J. Singh and C. Fiolhais, *Phys. Rev. B*, 1993, **48**, 4978-4978.
7. D. Vanderbilt, *Phys.Rev.B*, 1990, **41**, 7892.
8. K. Laasonen, R. Car, C. Lee and D. Vanderbilt, *Phys. Rev. B*, 1991, **43**, 6796.
9. Z. G. Yu, H. Gong and P. Wu, *J. Cryst. Growth*, 2006, **287**, 199-203.
10. H. Hellmann, *J. Chem. Phys.*, 1935, **3**, 61-61.
11. K. Laasonen, A. Pasquarello, R. Car, C. Lee and D. Vanderbilt, *Phys. Rev. B*, 1993, **47**, 10142.
12. C. Lee, D. Vanderbilt, K. Laasonen, R. Car and M. Parrinello, *Phys. Rev.B*, 1993, **47**, 4863.
13. W. H. Press, W. Vetterling, S. A. Teukolsky, B. P. Flannery and E. Greenwell Yanik, *SIAM Review*, 1994, **36**, 149-149.
